# Activin-A and Bmp4 Levels Modulate Cell Type Specification during CHIR-Induced Cardiomyogenesis

**DOI:** 10.1371/journal.pone.0118670

**Published:** 2015-02-23

**Authors:** Min-Su Kim, Audrey Horst, Steven Blinka, Karl Stamm, Donna Mahnke, James Schuman, Rebekah Gundry, Aoy Tomita-Mitchell, John Lough

**Affiliations:** 1 Department of Cell Biology, Neurobiology and Anatomy, Medical College of Wisconsin, Milwaukee, Wisconsin, United States of America; 2 Department of Surgery, Division of Cardiothoracic Surgery, Medical College of Wisconsin, Milwaukee, Wisconsin, United States of America; 3 Department of Biochemistry, Medical College of Wisconsin, Milwaukee, Wisconsin, United States of America; 4 The Cardiovascular Center, Medical College of Wisconsin, Milwaukee, Wisconsin, United States of America; National Cancer Center, JAPAN

## Abstract

The use of human pluripotent cell progeny for cardiac disease modeling, drug testing and therapeutics requires the ability to efficiently induce pluripotent cells into the cardiomyogenic lineage. Although direct activation of the Activin-A and/or Bmp pathways with growth factors yields context-dependent success, recent studies have shown that induction of Wnt signaling using low molecular weight molecules such as CHIR, which in turn induces the Activin-A and Bmp pathways, is widely effective. To further enhance the reproducibility of CHIR-induced cardiomyogenesis, and to ultimately promote myocyte maturation, we are using exogenous growth factors to optimize cardiomyogenic signaling downstream of CHIR induction. As indicated by RNA-seq, induction with CHIR during Day 1 (Days 0–1) was followed by immediate expression of Nodal ligands and receptors, followed later by Bmp ligands and receptors. Co-induction with CHIR and high levels of the Nodal mimetic Activin-A (50–100 ng/ml) during Day 0–1 efficiently induced definitive endoderm, whereas CHIR supplemented with Activin-A at low levels (10 ng/ml) consistently improved cardiomyogenic efficiency, even when CHIR alone was ineffective. Moreover, co-induction using CHIR and low levels of Activin-A apparently increased the rate of cardiomyogenesis, as indicated by the initial appearance of rhythmically beating cells by Day 6 instead of Day 8. By contrast, co-induction with CHIR plus low levels (3–10 ng/ml) of Bmp4 during Day 0–1 consistently and strongly inhibited cardiomyogenesis. These findings, which demonstrate that cardiomyogenic efficacy is improved by optimizing levels of CHIR-induced growth factors when applied in accord with their sequence of endogenous expression, are consistent with the idea that Nodal (Activin-A) levels toggle the entry of cells into the endodermal or mesodermal lineages, while Bmp levels regulate subsequent allocation into mesodermal cell types.

## Introduction

In order to utilize human pluripotent-derived cells for cardiac disease modeling, drug testing and therapeutics, protocols are required that reproducibly and efficiently induce cardiomyogenesis, ultimately resulting in homogeneous populations of differentiated cardiomyocytes. Fulfillment of this outcome requires the efficient induction of mesoderm, followed by segregation of cells into the cardiovascular and ultimately cardiomyogenic lineages, beginning at the earliest stages of pluripotent cell induction. Whereas protocols utilizing direct growth factor application have yielded context-dependent success [1], the use of small molecular weight (MW) organic molecules to modulate Wnt signaling, based on its regulation of early embryonic development [2–4] and cardiomyogenesis in mouse [4,5] and human [6] embryonic stem cells (ESCs), was recently shown to induce cardiomyogenesis in various pluripotent cell-lines maintained in monolayer format [7,8]. In the latter protocol, pluripotent cells are sequentially treated with CHIR and IWP, in the absence of exogenous growth factors, to induce then subsequently inhibit Wnt signaling; alternative small MW modulators of Wnt signaling are also effective [9]. Most recently, this approach has been improved via the design of minimal chemically-defined media [10]. Despite these remarkable advances, protocol adjustments that improve the reproducibility, rate and maturation of cardiomyogenesis induced by small MW modulators such as CHIR are warranted.

We have begun to address this objective by testing the hypothesis that cardiomyogenesis can be improved by optimizing levels of CHIR-induced growth factors during the earliest stages of induction from pluripotent cells, as mesoderm and its subtypes become specified; to efficiently induce cardiomyogenesis, mesoderm rather than endoderm must first be specified, followed by the specification of mesodermal sub-types. We employed RNA-seq to identify growth factors and cognate receptors induced by CHIR in monolayered pluripotent human H1 ESCs. Among the pathways induced, ligand and receptor components of Nodal and Bmp signaling respectively peaked during Days 1–3 and Days 3–14 of the differentiation period. To improve CHIR-induced cardiomyogenesis we have modulated these pathways, examining the effect of augmenting CHIR with the Nodal mimetic Activin-A, or with Bmp4, during the first day of induction. This revealed that co-induction with CHIR supplemented with high levels (50–100 ng/ml) of Activin-A efficiently induced definitive endoderm (DE), whereas supplementation with 10 ng/ml (i.e. low) Activin-A enhanced the reproducibility, and apparent rate, of cardiomyogenesis in comparison with cells induced with CHIR alone. Remarkably, inclusion of low Activin-A induced cardiomyogenesis in instances when CHIR alone was minimally effective; moreover, supplementation of CHIR with low Activin-A during Day 0–1 consistently induced the appearance of rhythmically beating cells by Day 6 rather than Day 8. In contradistinction to the pro-cardiomyogenic effect of inducing pluripotent cells with CHIR and low Activin-A, co-induction with CHIR and 3–10 ng/ml Bmp4 inhibited cardiomyogenesis, inducing robust expression of FOXF1, a posterior mesoderm marker, followed by the appearance of vesicular structures that exhibited no cardiomyogenic traits; by contrast, withholding Bmp4 until Days 3–5 either had no effect, or modestly enhanced cardiomyogenesis. These findings demonstrate that CHIR-modulated cardiomyogenesis can be enhanced by augmenting downstream signaling with exogenous growth factors, as in the instance of low Activin-A, when applied at optimal levels and in accord with their sequence of endogenous expression. Conversely, the inhibitory effect of exogenous BMP, low levels of which are required for mesoderm formation, implies that suppression of endogenous signaling induced by CHIR may be required in some instances. These findings are also consistent with the idea that Nodal (Activin-A) levels regulate differentiation into endodermal versus mesodermal germ layers, while Bmp levels regulate the subsequent specification of mesodermal cell types.

## Materials and Methods

Detailed methods are described in **S1 Methods.**

### Cells & Reagents

Human embryonic stem cell (hESCs) lines H1 (WA01) and H9 (WA09), were purchased from the National Stem Cell Bank (NSCB; WiCell, Madison WI). Human NKX2-5(eGFP/w) ESCs were a gift from Professors A.G Elefanty and E.G. Stanley, Monash University [11]. Human induced pluripotent iPSK3 (K3) cells were a gift from Professor Stephan A. Duncan of this institution [12]. Human iPSC line 963 was provided by the Wanek Consortium for HLHS (Mayo Medical School and Children’s Hospital of Wisconsin). All experiments utilizing H1 cells were performed during passages 30–70. Activin-A (338-AC-005), Bmp4 (314-BP-010), and Wnt3a (5036-WN-010) were from R&D Systems. Fgf2 (bFgf) was from Invitrogen (PHG0026). The small MW organic inhibitors employed in these experiments were CHIR99021 (Stemgent 04-0004-2) and IWP2 (Tocris 3533). Primary antibodies employed for immunofluorescent staining included anti-Myosin Heavy Chain monoclonal (DSHB MF20); anti-Oct4 monoclonal (Chemicon MAB-4305) or rabbit polyclonal (Santa Cruz sc-9081); and anti-Sox17 goat polyclonal (R&D Systems AF1924). Antibodies for flow cytometry were monoclonal anti-cardiac Cardiac Troponin T (TNNT2; Thermo Scientific MS-295-R7) coupled with secondary goat anti-mouse 594 IgG1 (Invitrogen #A21125); Alexa Fluor 488-conjugated mouse myeloma IgG MOPC-21 (Thermo Scientific 557721) was used as an IgG1 isotype control.

### Cardiomyocyte Differentiation

The experiments described in S1 and S2 Figs. employed pluripotent cells grown in hESC medium pre-conditioned by MEFs (MEF-cm) containing fresh bFGF (4 ng/ml) on E-cadherin substrate; to induce differentiation, pluripotent cells were expanded to ∼125% confluence, followed by exchanging medium for RPMI Medium 1640 (Invitrogen 11835-030) supplemented with B27 (Invitrogen 0050129SA) without insulin plus the indicated growth factors. All other experiments were performed as previously described [7], except that pluripotent cells were kept in an hypoxic (4% O_2_) environment. Briefly, pluripotent cells were plated in monolayer within 35 mm cell culture dishes coated with Matrigel substrate, in mTeSR1 medium. At Day -3 or Day -1, the cells were re-coated with Matrigel during medium change, to a final density of 8 μg/cm^2^. Prior to inducing differentiation, pluripotent cells were allowed by become super-confluent (∼125%). Differentiation was induced on Day 0 by moving the cultures to a normoxic environment and exchanging mTeSR1 medium for RPMI/B27 without insulin, supplemented with 12 μM (lot #2721) or 7.5 μM (lot #2914) CHIR99021 (Stemgent 04-0004-2; in our experience, CHIR efficacy is lot-dependent). After 20 hours (Day +1) the medium was replaced with RPMI/B27 medium without insulin and CHIR. On Day +3 the medium was exchanged for RPMI/B27 without insulin, with 5 μM IWP (Tocris 3533). Two days later (Day +5) the medium was replaced with RPMI/B27 without insulin and without inhibitor. At Day +7, the medium was changed to 2.0 ml RPMI/B27 with insulin, followed by identical medium changes at 2 day intervals thereafter. Experiments were terminated at Day +14, when cultures were evaluated for percentages of cardiac troponin-T-positive (cTnT+) cells using flow cytometry, which was performed on cells isolated from duplicate dishes and correlated with immunostaining of α-myosin heavy chain (αMHC; MF-20) in parallel cultures.

### Quantitative RT/PCR

Realtime PCR was performed as described in **S1 Methods.**

### RNA-seq

RNA-sequencing libraries were prepared using mRNA purified from duplicate 35 mm dishes on Days 0, 1, 3, 5, 8 and 14 of the induction period. Details are provided in the **S1 Methods**. This RNA-seq dataset has been deposited in the NIH Short Read Archive (NCBI SRA), accession number SRP048993.

### Data Analysis

Experimental variability and statistical significance was determined as indicated in each figure legend.

## Results

### High Levels of Activin-A Induce Sox17 in H1 ESCs

In initial experiments we attempted to specify the cardiomyogenic lineage in monolayered H1 cells using sequential or simultaneous treatment with Activin-A and Bmp4 [13]. With notable exceptions [14], success was limited, and could not be improved by modifications including the induction of cells in high density multilayer or by maintaining them on E-cadherin rather than Matrigel substrate (not shown). Because Wnt had been shown to regulate primitive streak formation [2,3] and to induce cardiomyogenesis in mouse [4,5] and human [6] ESCs, monolayered pluripotent cells were induced with Activin-A, Bmp4 and Wnt3a, alone and in combination. Induction with Bmp4 alone consistently induced a low monolayer of contiguous cells that exhibited Oil Red O-positive vesicles (S1 Fig.), possibly representing trophoblast cells as previously described [15,16]. Cells induced with Wnt3a alone were not viable (not shown). However, co-induction with Wnt3a, Bmp4, and increasing levels of Activin-A during Day 0–1 revealed that although all levels of Activin-A induced brachury (T; S2 A-C Fig.) and low levels of Sox17 (S2 D-F Fig.) during Day 1, high levels of Activin-A (50–100 ng/ml) induced very high expression of the endoderm marker Sox17 by Day 3 (S2F Fig.), indicating induction of definitive endoderm (DE). Conversely, cells treated with a low level (10 ng/ml) of Activin-A during Day 0–1 immediately expressed MESP1 (S2G Fig.), implying that low Activin-A induces the cardiomyogenic lineage in monolayered H1 cells, as previously reported for cells grown in embryoid body (EB) format [17,18].

### Efficient Induction of Cardiomyogenesis using Wnt Modulation with Matrigel Overlay

**Fig 1:**
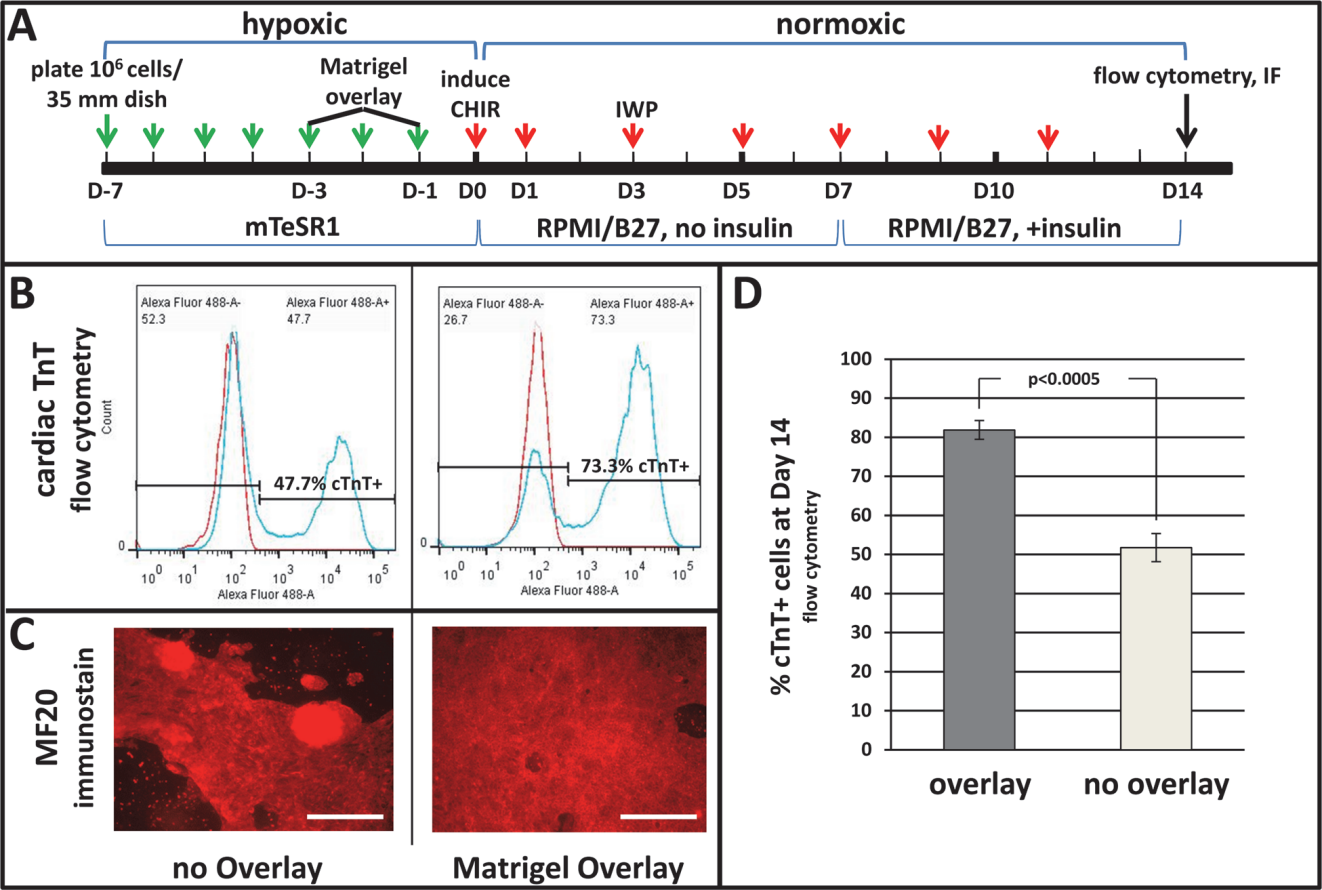
Wnt-modulated cardiomyogenesis is enhanced by Matrigel overlay. Pluripotent H1 ESCs expanded on Matrigel in mTeSR1 medium were treated with Matrigel overlays on Day -3 or -1. Differentiation was induced by changing medium to RPMI/B27 (no insulin) containing the small MW inhibitors CHIR (12 μmol/L) during Day 0–1 and IWP (5 μmol/L) during Days 3–5; insulin (4,000 ng/ml) was included after Day 7. **A**, scheme of cardiomyogenic induction using Matrigel overlay and small MW inhibitors; arrows denote days when medium was changed, before (green) and after (red) induction. **B**, typical flow cytometry results showing percentages of cardiac troponin-T (cTnT)-positive cells at Day 14. **C**, αMHC (MF20) immunostaining at Day 14. **D**, bar graph showing averaged flow cytometry results obtained in three independent determinations. Vertical lines depict ± SEM. Size bars in C = 200 μm. The p-value in D was calculated using Student’s t-test.

As this work was in progress it was reported that low MW organic inhibitors—CHIR and IWP—that modulate Wnt signaling could induce cardiomyogenesis in the absence of exogenous growth factor proteins [7]. As shown in Fig. 1 we verified this protocol (henceforth the ‘2-inhibitor protocol’), showing in addition that re-application of Matrigel to the expanding pluripotent cell monolayer, as previously shown for cells induced with Activin-A and Bmp [19], substantially enhanced differentiation as indicated by increased percentages of cardiac troponin-T-positive cells (Fig. 1B,D) as well as by the extensive area of the monolayer occupied by αMHC-positive cells (Fig. 1C) at Day 14. Identity of these cells as cardiomyocytes was indicated by their rhythmic contractions, which progressed from isolated sites at Day 8 to include the entire dish by Day 14, and by the exhibition of sarcomeres, which were not detected at these stages but were ultimately detectable in cultures maintained for 60 days (S3 Fig.).

### RNA-seq Reveals Endogenous Pathways that Regulate Mesoderm Formation and Cardiomyocyte Differentiation

**Fig 2:**
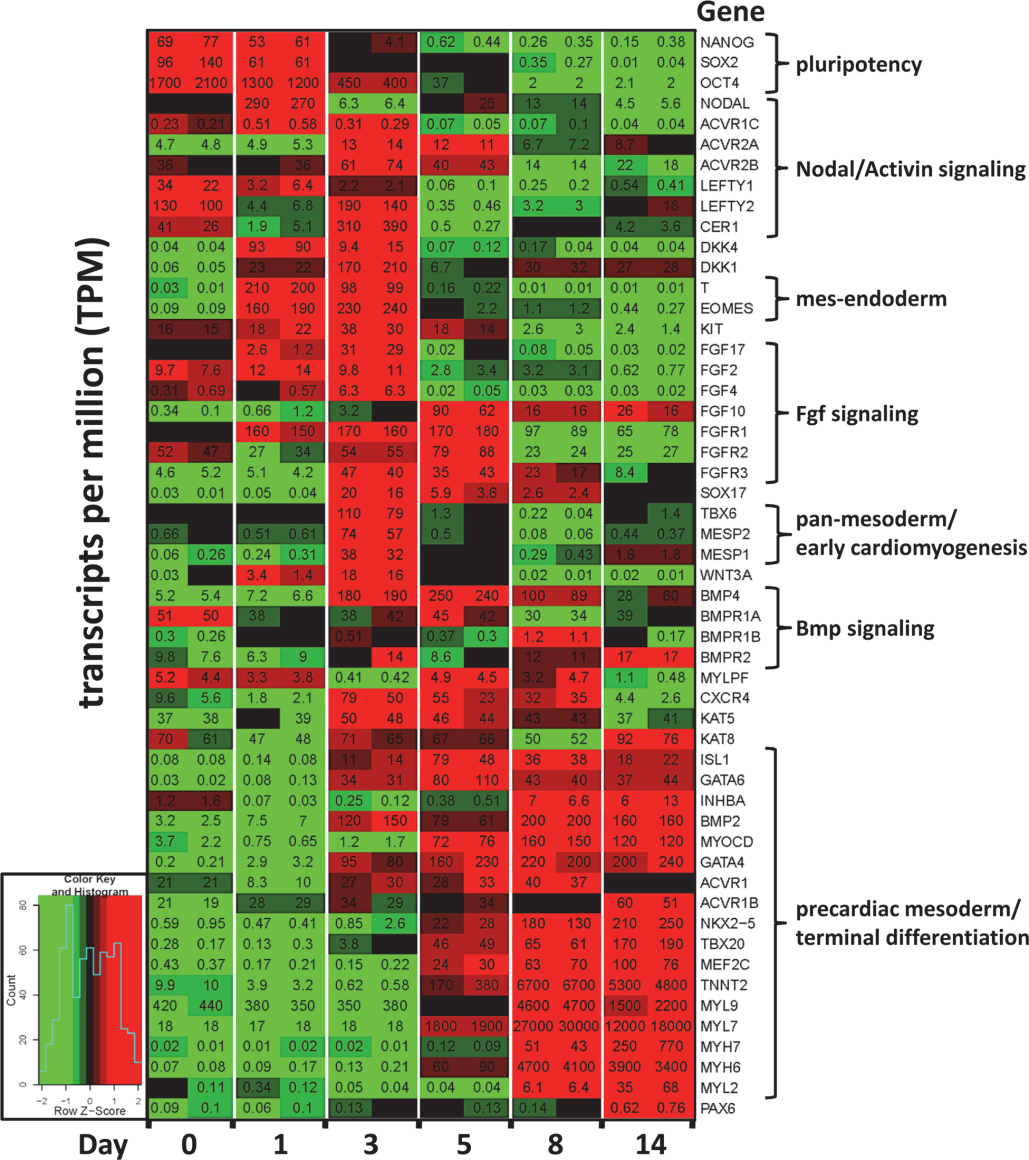
RNA-seq heat map revealing endogenous gene expression patterns during cardiomyogenesis induced with CHIR alone. Pluripotent H1 ESCs expanded on Matrigel in mTeSR1 medium were induced to differentiate by changing the medium to RPMI/B27 (without insulin) including the small MW inhibitors CHIR (12 μmol/L) during Day 0–1 and IWP (5 μmol/L) during Days 3–5; insulin (4,000 ng/ml) was included after Day 7. RNA was purified from duplicate cultures on the indicated days, converted to cDNA, and processed to RNA-seq libraries as described in **S1 Methods**. Colors indicate the range of each gene’s expression during the 14 day period, with least expression shown in green and highest expression shown in red (see inset). The number in each panel indicates the expression of each gene in transcripts per million (TPM) within each culture dish.

Although the 2-inhibitor protocol consistently induced cardiomyogenic cells, efficiency was variable, resulting in 5–85% cTnT-positive cells from experiment to experiment (not shown). We hypothesized that reproducible efficiency, as well as acceleration, could be improved by optimizing endogenous signaling downstream of CHIR-induced Wnt induction. To identify signaling pathways downstream of CHIR induction, the expression of genes in cells harvested from duplicate cultures on Days 0, 1, 3, 5, 8 and 14 of a 14 day differentiation period was analyzed by RNA-seq, selected results from which are shown in Fig. 2. In this particular determination, CHIR alone induced rhythmic beating that was first noted in isolated areas of the culture dishes at Day 8, and, 76% cTnT-positive cells were attained at Day 14 as assessed by flow cytometry. Regarding the Nodal/Activin-A pathway, CHIR-induction was immediately followed by peak expression of the primitive streak markers T (Fig. 2) and NODAL (Fig. 2 & S4A Fig.) during Day 1. This was followed by strong expression of the Activin type 2 receptor 2B (ACVR2B), with a less robust peak of ACVR2A (Fig. 2 & S4B Fig.); expression of these receptor subunits was confirmed in an alternate pluripotent cell-line by qPCR (S4B Fig., right panel), revealing that these were most detectably expressed only on Days 2 and 3 of the induction period. Expression of the Activin type 1 receptor ACVR1C (ALK7, Fig. 2, S4C & S5 Figs.) was highest during the early stages of induction (Days 0–3), while expression of the type 1 receptors ACVR1 and ACVR1B, which was higher, did not appreciably change during Days 0–14 (Fig. 2, S5 Fig.).

Regarding Bmp components it was surprising that the genes encoding ligands 2 and 4, which have been employed here [14] and by others [20] to induce cardiomyogenesis, did not become strongly expressed until Day 3, remaining high thereafter (Fig. 2, S4A Fig.). Regarding genes that encode Bmp receptors, BMPR1A was highly expressed throughout the induction period (S4C Fig.), while BMPR2 slowly increased throughout the 14 day induction period (S4B Fig.). BMPR1B was weakly expressed at all times (S4C Fig.).

### Early Exposure to Activin-A (Day 0–1) Modulates Specification of Endoderm or Mesoderm, in Concentration-Dependent Fashion

**Fig 3:**
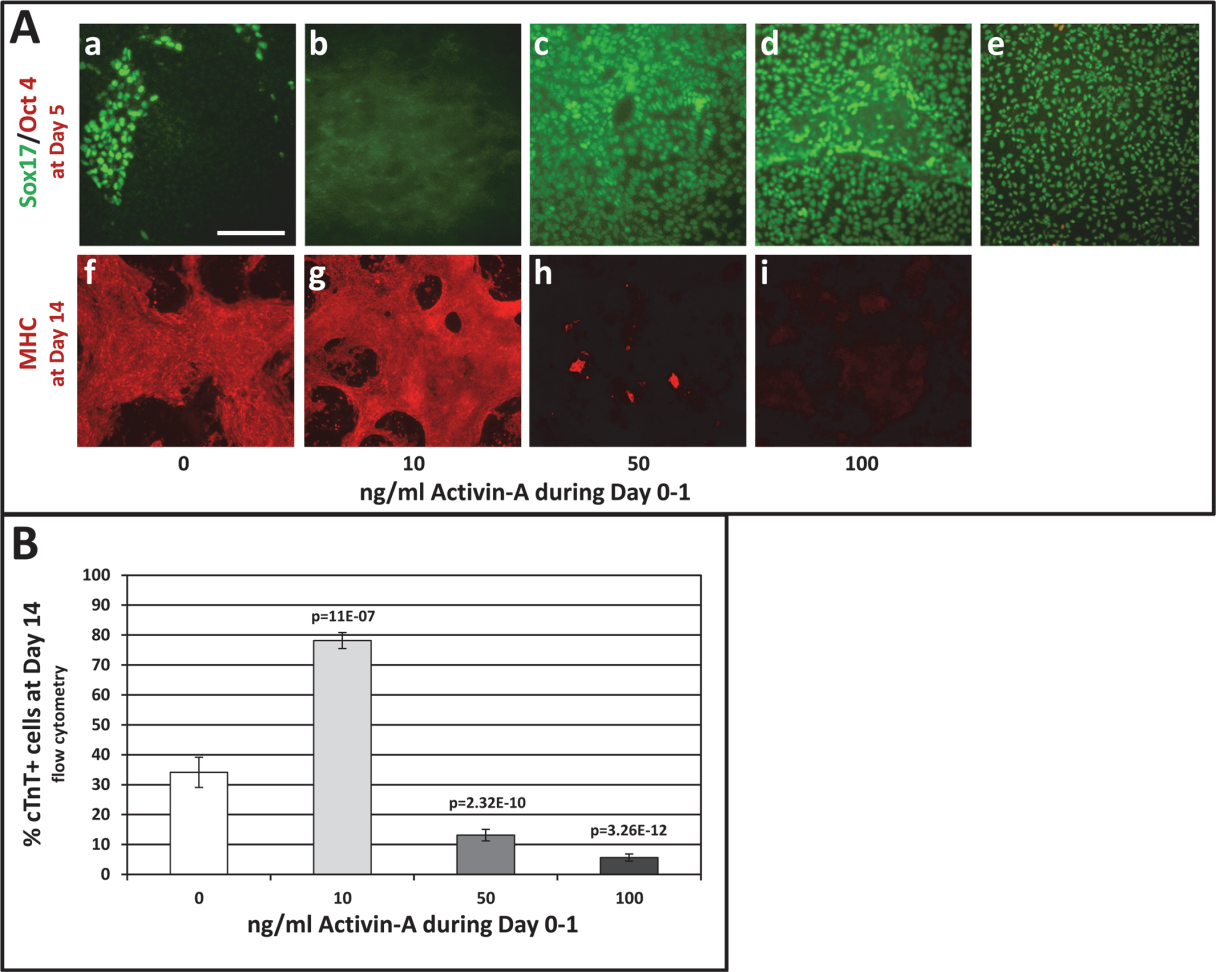
Activin-A levels during Day 0–1 modulate CM vs. DE differentiation. Pluripotent H1 ESCs were induced by changing medium to RPMI/B27 (no insulin), including CHIR (7.5 μmol/L) during Day 0–1 and IWP (5 μmol/L) during Days 3–5. Activin-A was included at the indicated levels during Day 0–1. Insulin (4,000 ng/ml) was included after Day 7. **Panel A**, a-e shows cells double-immunostained on Day 5 for Oct4 (red) and Sox17 (green); **e** is a positive control wherein cells were induced to DE with Activin-A (50 ng/ml) and Bmp4 (10 ng/ml) during Days 0–5. **Panel A f-i** shows cells immunostained with MF20 mAb on Day 14 to detect cardiomyocytes. **Panel B** depicts the effect of Activin-A levels during Day 0–1 on cardiomyocyte differentiation at Day 14, determined by flow cytometry using anti-cTnT. Cultures treated with 10 ng/ml Activin-A began to rhythmically contract at Day 6. Cells treated with 50 or 100 ng/ml Activin-A did not beat at any time. Bars indicate the average values combined from multiple experiments. Vertical lines = ±SEM. P-values were calculated by Student’s t-test. The p-value over the bar denoting 10 ng/ml Activin-A is relative to cells treated with CHIR only (0 ng/ml Activin-A), whereas the p-values over the bars denoting 50 and 100 ng/ml Activin-A are relative to cells treated with 10 ng/ml Activin-A. The size bar in Aa, which pertains to panels a-i, = 200 μm.

An early regulatory role for Activin-A signaling was indicated by expression peaks for NODAL (Fig. 2 & S4A Fig.) and the Activin receptor ACVR1C (S4C Fig.) immediately after CHIR induction (Day 1), followed at Day 3 by expression peaks of ACVR2A and ACVR2B (S4B Fig.), which ligand-binding receptors transduce the Nodal signal in the embryo [21]. Expression of the Nodal inhibitors LEFTYA and CERBERUS (CER1) at Day 3 (Fig. 2) suggested a requirement to moderate Nodal/Activin signaling. These data, plus our observations including those described in S2 Fig. indicating that high Activin-A levels promoted DE differentiation, prompted the experiments shown in Fig. 3 wherein pluripotent cells were induced during Day 0–1 with CHIR plus low or high levels of Activin-A, predicting respective cardiomyogenic or endodermal outcomes. As expected, high levels of Activin-A (50–100 ng/ml) induced DE, as indicated by expression of Sox17 in nearly all cells by Day 5 (Fig. 3A,c,d). High levels of Activin-A during Day 0–1 abolished cardiomyogenesis, as indicated by the absence of MHC immunostaining (Fig. 3A h-i), a result corroborated by the absence of beating throughout the culture period and by low percentages of cTnT-positive cells at Day 14 (Fig. 3B). By contrast, cells co-induced with a low level (10 ng/ml) of Activin-A displayed significantly increased percentages of cTnT-positive cells at Day 14 (Fig. 3B), which was corroborated by widespread expression of αMHC as shown by low magnification images of parallel cultures that were immunostained with MF20 antibody (Fig. 3A,g). The pro-cardiomyogenic effect of Activin-A was most pronounced in experiments wherein CHIR alone was relatively ineffective; for example, in one experiment where CHIR alone failed to induce beating or cTnT-positive cells by Day 10, cells that had been co-induced with CHIR plus low Activin-A during Day 0–1 exhibited strong and timely expression of early lineage markers, followed by the onset of beating at Day 6 and differentiation of ∼85% cTnT positive cells at Day 10 (S6 Fig.). Remarkably, whereas cells induced with CHIR only have never been observed to beat before Day 8, cells induced with CHIR and 10 ng/ml Activin-A during Day 1 began to exhibit rhythmic beating at Day 6 in 25 of 29 instances.

### Early Exposure to Bmp4 (Day 0–1) Inhibits CHIR-Induced Cardiomyogenesis

**Fig 4:**
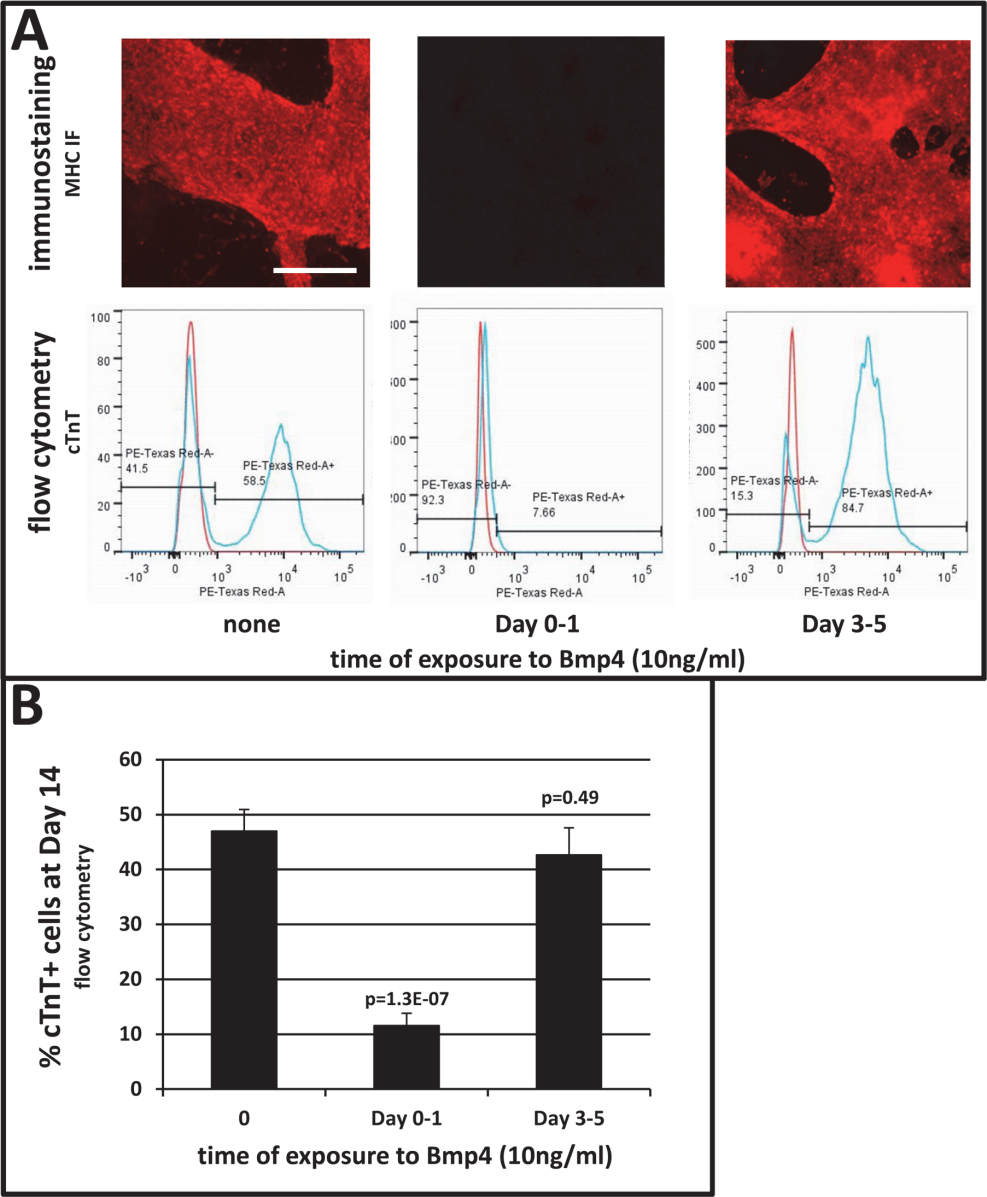
Treatment of Wnt-modulated cells with Bmp4 during Day 0–1 inhibits cardiomyogenesis. Pluripotent H1 ESCs were expanded and induced as described in Fig. 1. Cells induced by Wnt modulation were treated with Bmp4 (10 ng/ml) for the indicated durations. Cells treated with Bmp4 during Day 1 never beat, whereas cells treated during Days 3–5 were beating by Day 8. **A, upper**: αMHC immunostaining at Day 14. **A, lower**: cTnt flow cytometry of cells in parallel. **B**, percentages of cTnT+ cells assessed by flow cytometry at Day 14. Bars indicate the average of values combined from multiple experiments. Vertical lines = ±SEM. Each p-value is relative to cells treated with CHIR and IWP only (i.e. 0 ng/ml Bmp4). The size bar in A, which pertains to all immunostains in this panel, = 200 μm.

RNA-seq revealed that expression of BMP ligands 2 and 4 did not peak until Day 3 (Fig. 2, S4A Fig.), and that expression of the ligand-binding BMPR2 receptor was low at the earliest stages (Fig. 2, S4B Fig.), suggesting that augmentation of CHIR with Bmp2/4 during Day 0–1 should not substantially affect cardiomyogenesis. Pluripotent cells were induced with CHIR, with and without 10 ng/ml Bmp4, during Days 0–1. Cells treated with CHIR alone differentiated into cardiomyocytes, as revealed by rhythmic beating starting at Day 8 and widespread expression of αMHC (Fig. 4A) and cardiac TnT (Fig. 4A,B) at Day 14. However, cells co-induced with CHIR and Bmp4 did not differentiate into cardiomyocytes, as indicated by absence of beating throughout the 14 day culture period, the absence of αMHC immunostaining (Fig. 4A), and by significantly depressed percentages of cTnT-positive cells at Day 14 (Fig. 4B). Remarkably, in 21 of 21 cultures examined over several experiments, cells co-induced with CHIR and 10 ng/ml Bmp4 during Day 0–1 began patterning into three-dimensional vesicular structures at Day 7, which became widespread by Day 10 and remained prominent thereafter (S7A Fig.), indicating differentiation into non-cardiomyogenic cells. While the identity of these cells is unknown, qPCR revealed strong expression of the posterior streak marker FOXF1 (avg. >4x10^3^-fold) immediately after Bmp exposure (S8 Fig.), which was accompanied by significant expression of KDR (FLK1; not shown). A dose-response determination revealed that co-induction with CHIR and Bmp4 at levels as low as 3 ng/ml during Day 0–1 inhibited cardiomyogenic differentiation (S7B Fig.). By contrast, withholding Bmp4 until Days 3–5 usually had no appreciable effect (S7B,C Fig.), or in some instances induced modest increases in cTnT-positive cells at Day 14 (not shown).

The determinations described in Figs. 1–4 were performed using human H1 ESCs. While this line is considered to be a reliable standard, it was important to evaluate the effect of co-inducing with CHIR and Activin-A or Bmp4 in other pluripotent cell-lines. Determinations have been performed using two additional human ESC lines (H9 & NKX2-5e[GFP/w]), and two human induced pluripotent cell (iPSC) lines (963 & K3), as respectively shown in S9 and S10 Figs. Results are consistent with effects on H1 cells described in Figs. 3–4, i.e., low Activin-A promoted cardiomyogenesis, especially in instances when CHIR alone was minimally effective (S9A & S10B Figs.), while high Activin-A induced endoderm (S9A & S10A Figs.). And, 10 ng/ml Bmp4, when included with CHIR during cardiomyogenic induction, was inhibitory in all instances.

## Discussion

A goal of this and other laboratories is to establish conditions wherein human cardiomyocytes efficiently differentiate and ultimately mature in monolayer culture. Using protocols that utilize induction with Bmp and Activin-A, it was observed that extended treatment with the former induced cells resembling trophoblasts (S1 Fig.), while treatment with high levels of the latter during Day 0–1 induced high levels of the DE marker Sox17 (S2 Fig.). Because use of these factors was based on their ability to induce terminal differentiation in precardiac mesoderm explanted from post-gastrulation embryos [22], findings that Wnt, which regulates earlier stages of embryogenesis [2–4], induces the cardiomyogenic lineage [4–6] were not surprising. In our experience, induction of Wnt signaling using small MW inhibitors—CHIR and IWP—as described by the Palecek laboratory [8], rather than protein growth factors, most reliably induces cardiomyogenesis (Fig. 1). Nonetheless, variable outcomes, perhaps attributed to non-uniform effects of CHIR, caused us to address whether optimization of signaling downstream of Wnt-induced modulation can improve cardiomyogenic differentiation.

As shown in Fig. 1D, deployment of a Matrigel overlay prior to Wnt induction, as recently reported for cells induced with Activin-A/Bmp [19], significantly improved cardiomyogenic differentiation in H1 cells that were subjected to the 2-inhibitor protocol. However, efficiency remained inconsistent, for possible reasons including those discussed by the authors [19]. In addition, we have observed lot-to-lot, and even experiment-to-experiment, variability of CHIR efficacy. Therefore, with the goal of optimizing signaling downstream of induction by CHIR, RNA-seq was used to reveal the sequence and duration of endogenous gene expression during a high efficiency outcome. While acknowledging that RNA-seq data from a determination performed on a single cell-line should be cautiously interpreted, the experimental precision shown in Fig. 2, S4 and S5 Figs. revealing the expression of ligands and cognate receptors in the Activin-A, Bmp and other (not shown) signaling pathways prompted the determinations described in Figs. 3 and 4.

First, the effect of modulating Activin-A/Nodal signaling during Day 0–1 with exogenous Activin-A was assessed. Fig. 3 shows that, consistent with the results described in S2 Fig. as well as with previous findings in EBs [17,18], monolayered cells differentiated into either cardiomyocytes or DE as respectively driven by low or high levels of Activin-A. Remarkably, co-induction with CHIR and low (10 ng/ml) Activin-A during Day 0–1 accelerated the onset of beating from Day 8 to Day 6 in 25/29 cultures to date. Hence it will be interesting to determine whether expression peaks of lineage markers such as *TBX6* and *MESP1*, and/or terminal differentiation markers such as *NKX2-5*, occur prior to peaks induced by CHIR alone, and whether adjusting exposure to IWP affects this process. Also, exposure to CHIR plus low Activin-A during Day 0–1 significantly increased percentages of cTnT-positive myocytes at Day 14. Conversely, supplementation of CHIR during Day 0–1 with high Activin-A (50–100 ng/ml) promoted efficient DE differentiation while strongly inhibiting cardiomyogenesis. Although it is well-established that similarly high levels of Activin-A induce DE, prolonged exposure over several (3–6) days is required [23–27]. Hence the result in Fig. 3 is somewhat surprising because pluripotent cells were exposed to high Activin-A during only the first 20 hours of induction (i.e. Day 0–1), and also because this treatment was sufficient to overcome the pro-mesodermal effect of β-catenin. This result may be reconciled with cardiomyogenic protocols that employ high levels of Activin-A during Day 0–1 [13,28] by considering that DE cells that are directly induced by this treatment may subsequently induce cardiomyogenesis in indirect fashion [27,29,30], and/or that the effects of Activin-A are context-dependent, perhaps reflecting the presence of lower levels of endogenous ligand in some cell-lines [18]. Such possibilities should be considered when designing cardiomyogenic protocols.

Second, Bmp has been used to induce cardiomyogenesis in cells cultured in both monolayer [13,28] and EB [17,20,31,32] format. Because RNA-seq indicated that expression of Bmp ligands 2 and 4 did not substantially increase in CHIR-induced cells until Day 3, it was decided to compare the effect of treating CHIR-induced cells with Bmp4 during Days 0–1 and Days 3–5. As shown in Fig. 4, inclusion of 10 ng/ml Bmp4 during induction with CHIR (Day 0–1) strongly inhibited cardiomyogenesis. Although this result was unexpected based on the above citations, it is consistent with findings that relatively high levels of Bmp promote hematopoiesis [33], and that inhibitors of Bmp signaling promote beating in human and mouse EBs [34–37]. While these data are difficult to reconcile with a report that induction of pluripotent cells with Bmp4 for one day, followed by three days’ treatment with the Bmp inhibitor noggin, favors cardiomyogenesis over erythropoiesis [15], this may reflect down-regulation of Bmp signaling resultant from long-term exposure to noggin. It is speculated that positive cardiomyogenic outcomes following treatment with Bmp4 during Days 1–5 [6,13,19,28] reflects, in part, its omission during the first day of induction, and/or the inclusion of bFgf, which antagonizes Bmp during early stages of differentiation [38,39].

Previous experiments performed by others [7] employed small MW inhibitors to disrupt signal transduction induced by Bmp and Nodal, demonstrating that pathways induced by both ligands are required during the earliest stage (Day 0–1) of CHIR-induced cardiomyogenic differentiation; these findings were recently corroborated [10]. Considered with the results reported here, these findings indicate that while signaling induced by Activin-A and Bmp ligands is required at this early step, their levels must be carefully regulated in order to prevent differentiation into alternative lineages. Because recent reports indicate that endoderm [40] and mesoderm [41] become specified in Smads2/3-dependent fashion via recruitment of the histone de-methylase Jmjd3 to germline-specific genes, it is interesting to speculate that one more of these factors are differentially responsive to Activin-A levels, thereby mediating an endodermal or a mesodermal outcome. Moreover, these findings suggest that the reproducibility of cardiomyogenesis induced by CHIR, and perhaps the ultimate maturation of cardiomyocytes, can be enhanced by altering the concentration of endogenous ligands that induce other signaling pathways, in a fashion that complies with their temporal sequence of expression. We are accordingly investigating the effect of modulating other signaling pathways, as revealed by the RNA-seq database (Fig. 2), during the first week of CHIR-induced cardiomyogenesis.

## Supporting Information

S1 Fig Induction with Bmp4 alone induces trophoblast-like cells. Pluripotent H1 ESCs expanded on E-cadherin were subcultured onto Matrigel and induced to differentiate by changing medium to RPMI/B27 (without insulin) including Bmp4 (10 ng/ml). Medium was changed daily, including Bmp4, for the next 5 days, during which time the cells assumed the low contiguous monolayer shown in **Panel A** (phase-contrast image). Parallel dishes fixed and stained with Oil red O displayed positive inclusions (**Panel B**). This phenomenon was consistently observed during five experimental repetitions.
(TIF)

S2 Fig High levels of Activin-A during Day 1 induce DE. Pluripotent H1 ESCs were sub-cultured on Matrigel and induced by changing medium to RPMI/B27 (without insulin) including Activin-A (indicated levels) and Wnt3a (25 ng/ml) during Day 0–1, and Bmp4 (10 ng/ml) during Days 0–5. **Panels A-O** show expression of the indicated genes after induction as determined by qRT-PCR normalized to RPL13A expression, and to the level of each gene’s expression in pluripotent cells at Day 0. Bars/vertical lines indicate the mean/range of duplicate values; similar results were obtained in two experimental repetitions (i.e. three experiments total).
(TIF)

S3 Fig α-Actinin immunofluorescent staining of cardiomyocyte cultures at differentiation Day 60. Pluripotent H1 ESCs we maintained and induced to differentiate as described for Fig. 1. **Panels A** and **B** show two magnifications of cardiomyocytes derived from H1 ESCs at differentiation Day 60. These cells, which were rhythmically contracting by Day 10, possessed organized sarcomeres by Day 60 as prominently shown in **Panel B**. DAPI (blue)-stained nuclei are shown in B.
(TIF)

S4 Fig Quantitative expression of NODAL (Activin-A) and BMP signaling components during CHIR-induced cardiomyogenesis. **Panels A-C**, which are derived from the RNA-seq determination (Fig. 2), respectively show quantitative levels of transcripts encoding (**A**) NODAL and BMP ligands, (**B**) Activin and Bmp type 2 ligand-binding receptors, and, (**C**) Activin and Bmp type 1 receptors during the 14 day cardiomyogenic period. Each point represents RNA-seq performed on a sample from a 35 mm culture dish. The qPCR determination to the right of **panel B** was performed during CHIR-induced differentiation of an alternative pluripotent cell-line (DF6-9-9T iPSCs; WiCell); bars and vertical lines respectively denote the mean ±SEM of values from triplicate cultures.
(TIF)

S5 Fig Expression of Activin Type 1 receptors during CHIR-induced cardiomyogenesis. Each point represents RNA-seq performed on cells harvested from a single 35 mm culture dish.
(TIF)

S6 Fig Augmentation of CHIR with 10 ng/ml Activin-A induces cardiomyogenesis when CHIR alone is ineffective. Pluripotent H1 ESCs expanded on Matrigel in mTeSR1 medium were overlaid with Matrigel on Day -1 and induced during Day 0–1 by changing the medium to RPMI/B27 (no insulin) containing CHIR (12 μmol/L) only, CHIR plus 10 ng/ml Activin-A, or CHIR plus 100 ng/ml Activin-A, as indicated. The cultures were treated with IWP (5 μmol/L) during Days 3–5, and, insulin (4,000 ng/ml) was included after Day 7. **Left Panels**: qPCR-based expression of T (Brachury), TBX6, and MESP1 on the indicated days after induction, normalized to expression of RPL13A and to the level of each gene’s expression in pluripotent cells (Day 0). **Right Panel**: Flow cytometric determination of cardiomyogenic cell percentages at Day 10. In this experiment, cells induced with CHIR alone during Day 0–1 did not contract at any time, whereas cells induced with CHIR plus 10 ng/ml Activin-A began to rhythmically contract in localized areas at Day 6, which became widespread by Day 10. This determination was unusual in that cultures treated with CHIR plus 100 ng/ml Activin-A during Day 0–1 exhibited localized foci of contracting cells at Day 10. Vertical lines denote ranges of duplicate values; AA = Activin-A.
(TIF)

S7 Fig Concentration- and duration-dependent effects of early exposure to Bmp4 during CHIR-induced cardiomyogenesis. Pluripotent H1 ESCs were induced with CHIR and Bmp4 at the indicated concentrations/durations. **Panel A** shows Bmp4-induced three-dimensional vesicles (arrows) that begin to appear at Day 7. **Panels B-C**, percentages of cTnT-positive cells at Day 14. **Panel B** shows the effect of various Bmp4 concentrations applied during Day 0–1; the effect of treatment with 10 ng/ml during Days 3–5 is shown at right for comparison. **Panel C** shows the effect of various Bmp4 concentrations during Days 3–5. Vertical lines indicate the range of duplicate values in B, and ±SEM of triplicate values in C. The size bar in A = 200 μm.
(TIF)

S8 Fig Early treatment with Bmp4 induces FOXF1, a posterior marker. Pluripotent H1 ESCs were induced by changing medium to RPMI/B27 (without insulin) including the indicated factors during Day 0–1. Fold expression of FOXF1 (Y axis) was assessed qRT-PCR and normalized to RPL13A (loading control), and to the levels of these mRNAs in pluripotent cells at Day 0. Numbers in parentheses indicate numbers of cultures; bars/vertical lines indicate mean/±SEM. The p-values are relative to cells treated with CHIR alone.
(TIF)

S9 Fig Effect of augmenting CHIR-induced cardiomyogenic differentiation with Activin-A or Bmp4 during Day 1 in other ESC lines. H9 (**Panel A**) and NKX2-5(eGFP/w) (**Panel B**) ESCs were expanded and induced with CHIR, along with the indicated levels of Activin-A or Bmp4 during Day 0–1. **Panel A**: Inclusion of 10 ng/ml Activin-A during induction augmented induction by CHIR alone, the latter of which was relatively ineffective in this determination. Inclusion of 10 ng/ml Bmp4 or 100 ng/ml Activin-A inhibited cardiomyogenesis at Day 14; by Day 5, 100 ng/ml Activin-A induced DE, indicated by Sox17-positive cells (**sub-panel b**). Red fluorescence = αMHC (MF20) immunostaining; green fluorescence = Sox17 immunostaining. Bars represent the mean of triplicate determinations; vertical lines = ±SEM. The p-value is relative to cells induced with CHIR alone. **Panel B**: Each condition was evaluated in duplicate cultures. In this experiment, although 10 ng/ml Activin-A did not significantly improve the level of differentiation induced by CHIR alone (which was robust in this instance), co-induction with higher Activin-A levels caused cell death by Day 14 (not shown). Co-induction with 10 ng/ml Bmp4 inhibited CHIR-induced cardiomyogenesis.
(TIF)

S10 Fig Effect of augmenting CHIR-induced cardiomyogenic differentiation with Activin-A or Bmp4 during Day 1 in iPSC lines. Pluripotent 963 (**Panel A**) and K3 (**Panel B**) iPSCs were expanded and induced with CHIR, along with the indicated levels of Activin-A or Bmp4 during Day 0–1. **Panel A**: Although 10 ng/ml Activin-A did not significantly improve the already high level of differentiation (cTnT-positive cells) induced by CHIR alone in this experiment, co-induction with 100 ng/ml Activin-A inhibited cardiomyogenesis as indicated by reduced numbers of cTnT-positive cells and the absence of both beating and myosin heavy chain staining (**sub-panel e**) at Day 10; the latter was concomitant with a high incidence of Sox17-positive cells noted at Day 5 (**sub-panel b**). Co-induction with 10 ng/ml Bmp4 during Day 0–1 inhibited CHIR-induced cardiomyogenesis at Day 10. Red fluorescence = αMHC (MF20) immunostaining; green fluorescence = Sox17 immunostaining. In **panel B** (K3 iPSCs), inclusion of 10 ng/ml Activin-A during induction strongly augmented the effect of CHIR, which was relatively ineffective in this determination, whereas inclusion of 10 ng/ml Bmp4 inhibited cardiomyogenesis. In both panels, bars represent the mean of triplicate determinations; vertical lines = ±SEM. P values were calculated by Student’s t-test; P values are relative to cells induced with CHIR alone.
(TIF)

S1 Methods Detailed Materials & Methods. (DOCX)
