# Supplementary figures and images for: Activin-A and Bmp4 Levels Modulate Cell Type Specification during CHIR-Induced Cardiomyogenesis
Source: PLoS One. 2015 Feb 23;10(2):e0118670. doi: 10.1371/journal.pone.0118670 (PMC4338295; doi:10.1371/journal.pone.0118670)

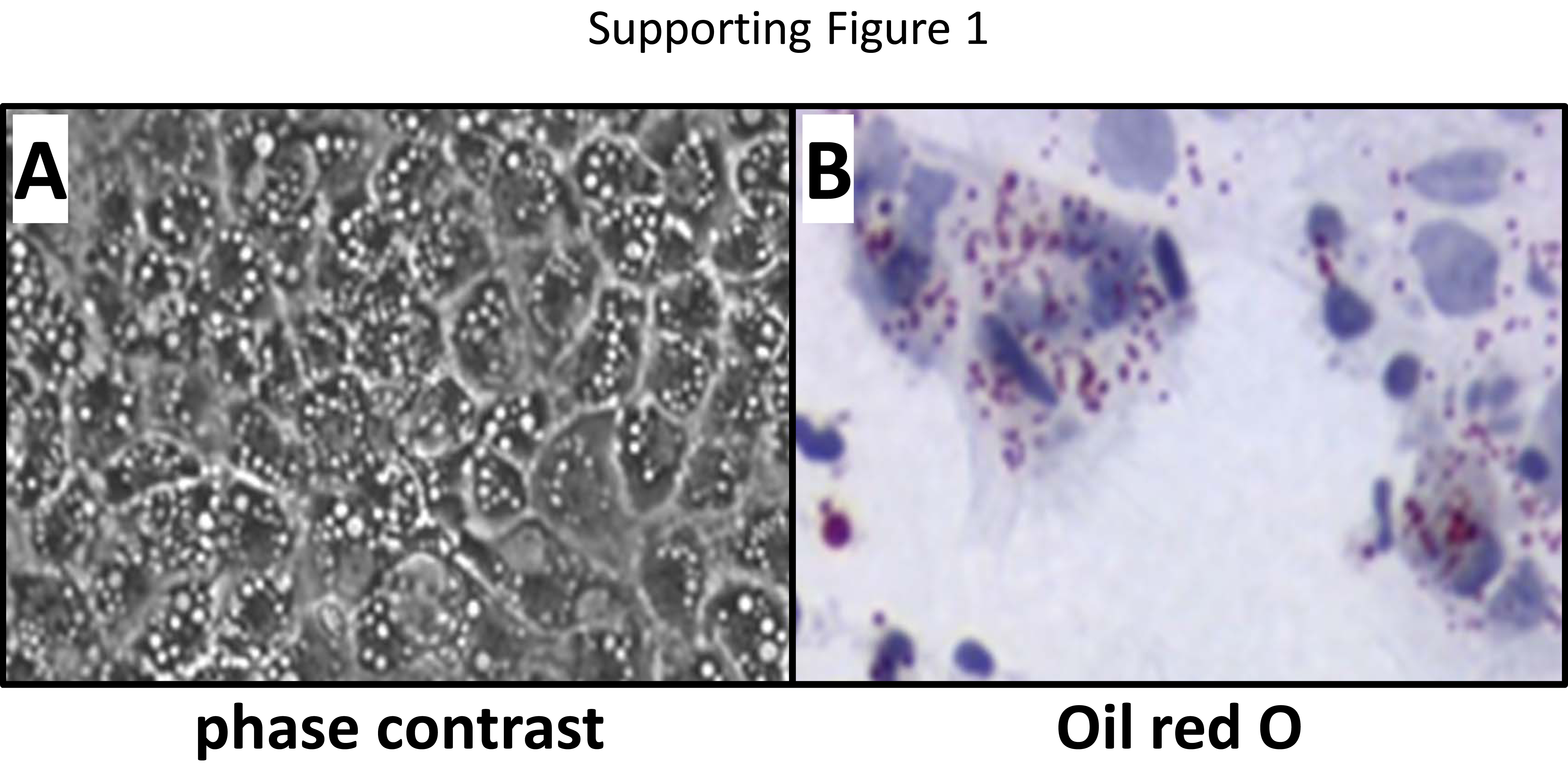

Supplement: S1 Fig — Pluripotent H1 ESCs expanded on E-cadherin were subcultured onto Matrigel and induced to differentiate by changing medium to RPMI/B27 (without insulin) including Bmp4 (10 ng/ml). Medium was changed daily, including Bmp4, for the next 5 days, during which time the cells assumed the low contiguous monolayer shown in Panel A (phase-contrast image). Parallel dishes fixed and stained with Oil red O displayed positive inclusions (Panel B). This phenomenon was consistently observed during five experimental repetitions. (TIF) [file pone.0118670.s001.tif]

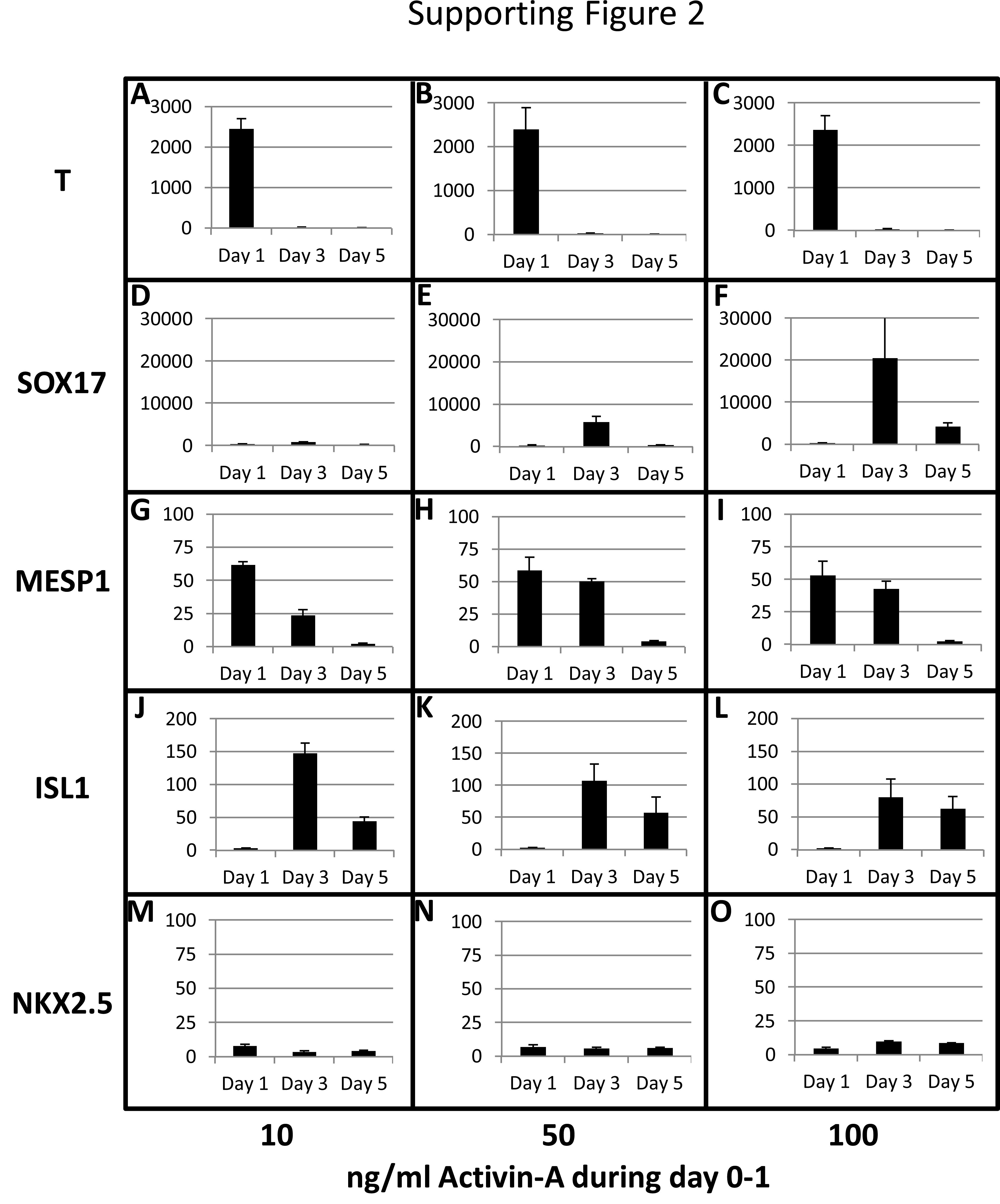

Supplement: S2 Fig — Pluripotent H1 ESCs were sub-cultured on Matrigel and induced by changing medium to RPMI/B27 (without insulin) including Activin-A (indicated levels) and Wnt3a (25 ng/ml) during Day 0–1, and Bmp4 (10 ng/ml) during Days 0–5. Panels A-O show expression of the indicated genes after induction as determined by qRT-PCR normalized to RPL13A expression, and to the level of each gene’s expression in pluripotent cells at Day 0. Bars/vertical lines indicate the mean/range of duplicate values; similar results were obtained in two experimental repetitions (i.e. three experiments total). (TIF) [file pone.0118670.s002.tif]

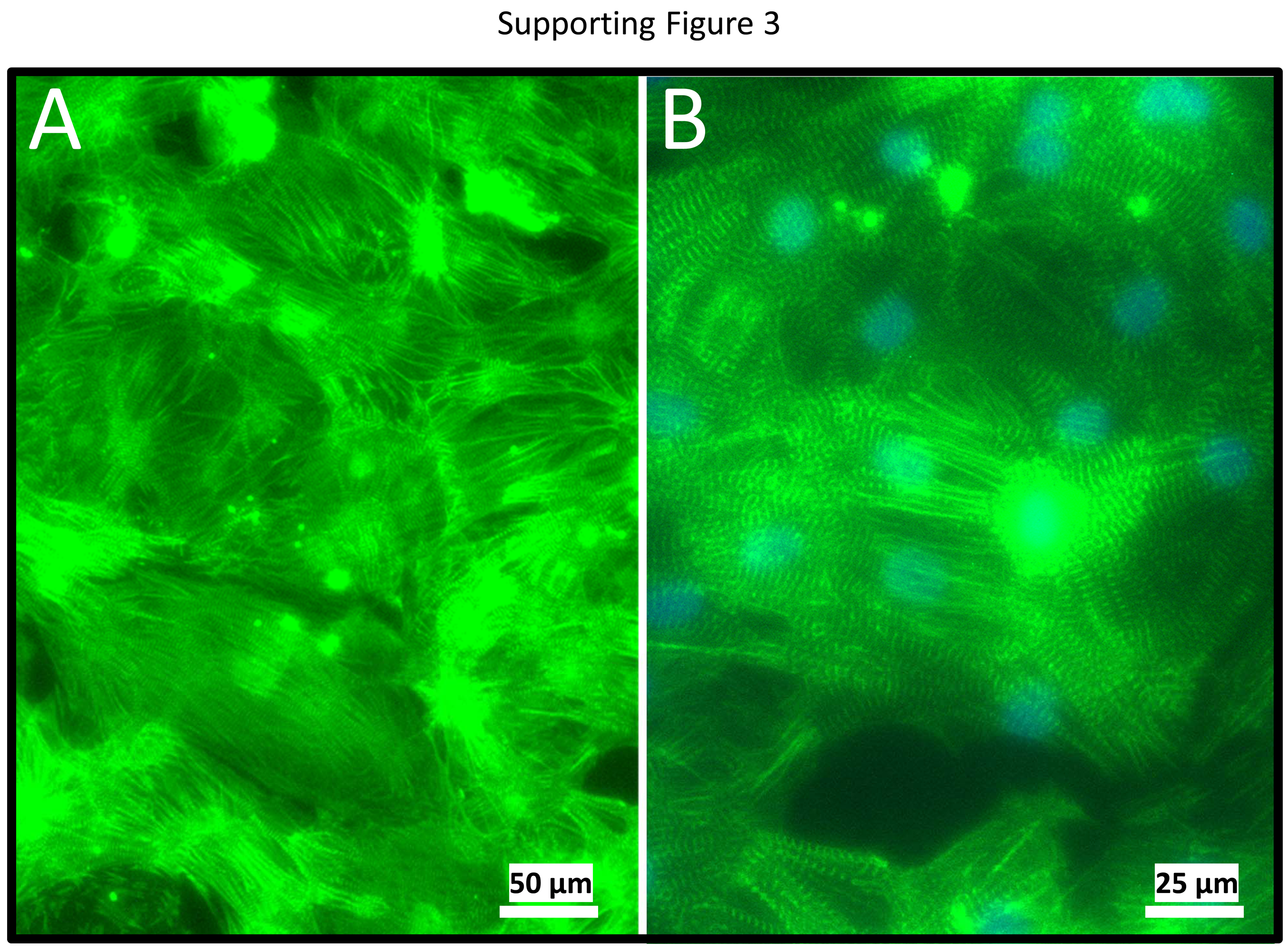

Supplement: S3 Fig — Pluripotent H1 ESCs we maintained and induced to differentiate as described for Fig. 1. Panels A and B show two magnifications of cardiomyocytes derived from H1 ESCs at differentiation Day 60. These cells, which were rhythmically contracting by Day 10, possessed organized sarcomeres by Day 60 as prominently shown in Panel B. DAPI (blue)-stained nuclei are shown in B. (TIF) [file pone.0118670.s003.tif]

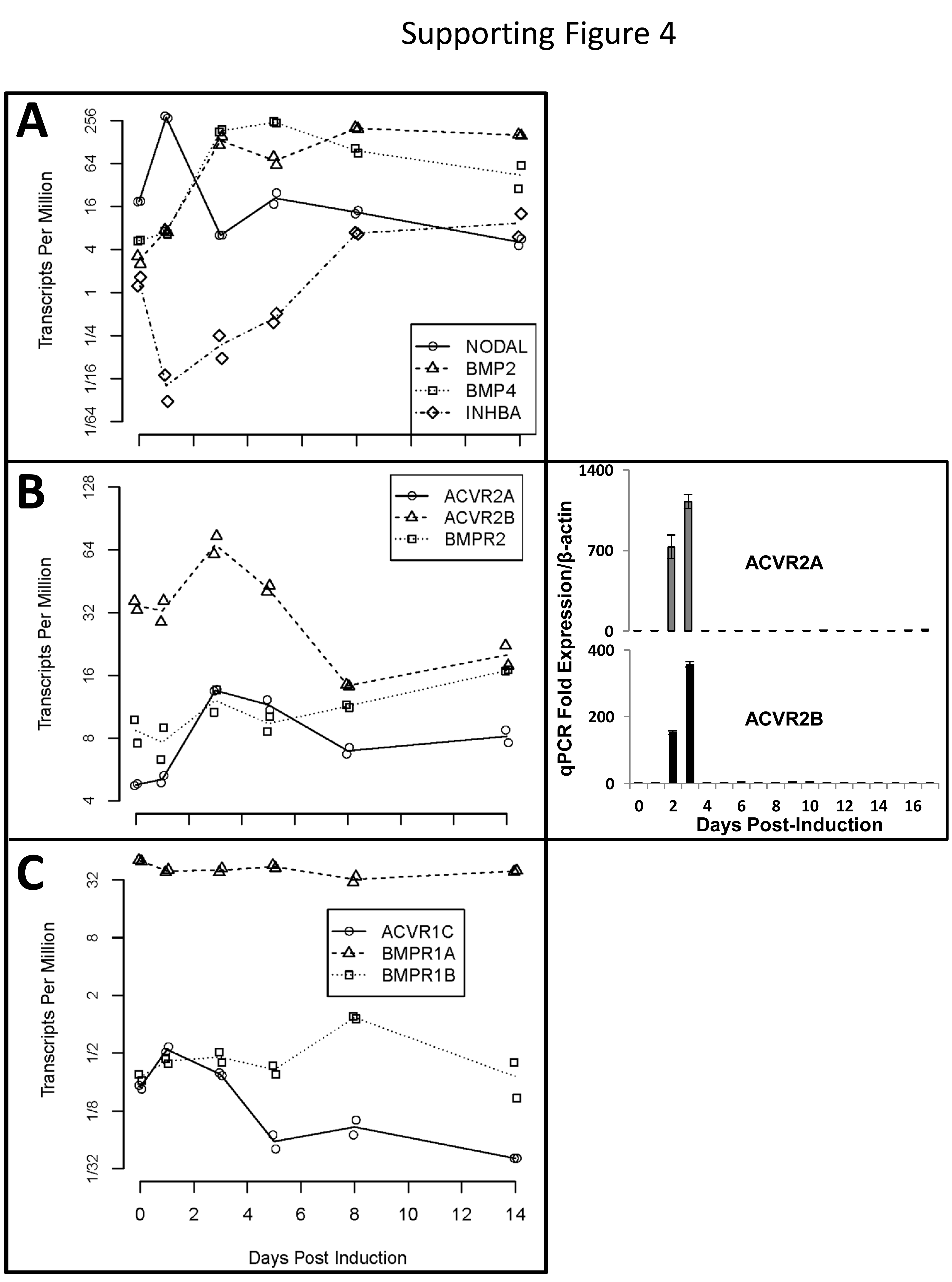

Supplement: S4 Fig — Panels A-C, which are derived from the RNA-seq determination (Fig. 2), respectively show quantitative levels of transcripts encoding (A) NODAL and BMP ligands, (B) Activin and Bmp type 2 ligand-binding receptors, and, (C) Activin and Bmp type 1 receptors during the 14 day cardiomyogenic period. Each point represents RNA-seq performed on a sample from a 35 mm culture dish. The qPCR determination to the right of panel B was performed during CHIR-induced differentiation of an alternative pluripotent cell-line (DF6-9-9T iPSCs; WiCell); bars and vertical lines respectively denote the mean ±SEM of values from triplicate cultures. (TIF) [file pone.0118670.s004.tif]

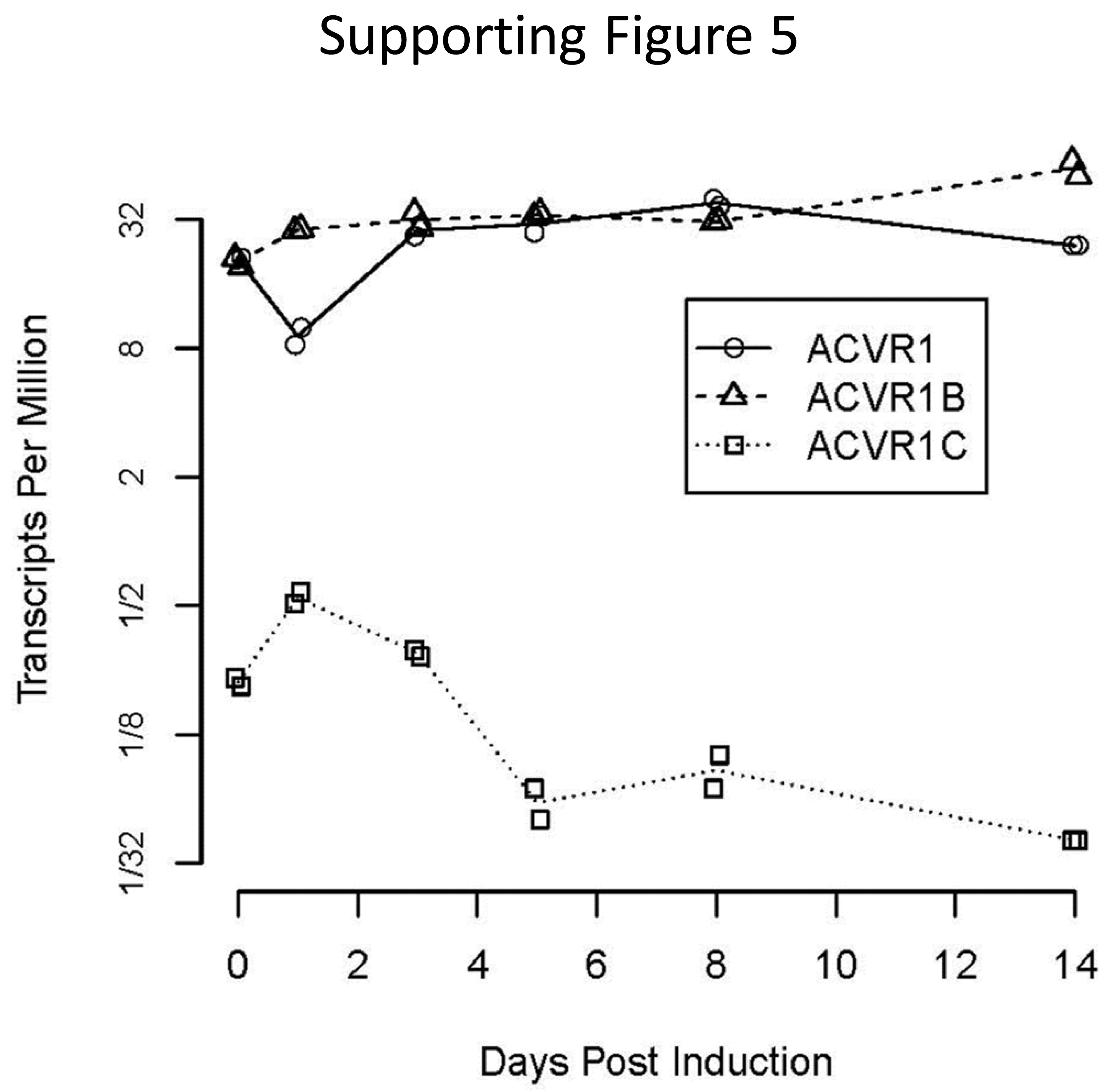

Supplement: S5 Fig — Each point represents RNA-seq performed on cells harvested from a single 35 mm culture dish. (TIF) [file pone.0118670.s005.tif]

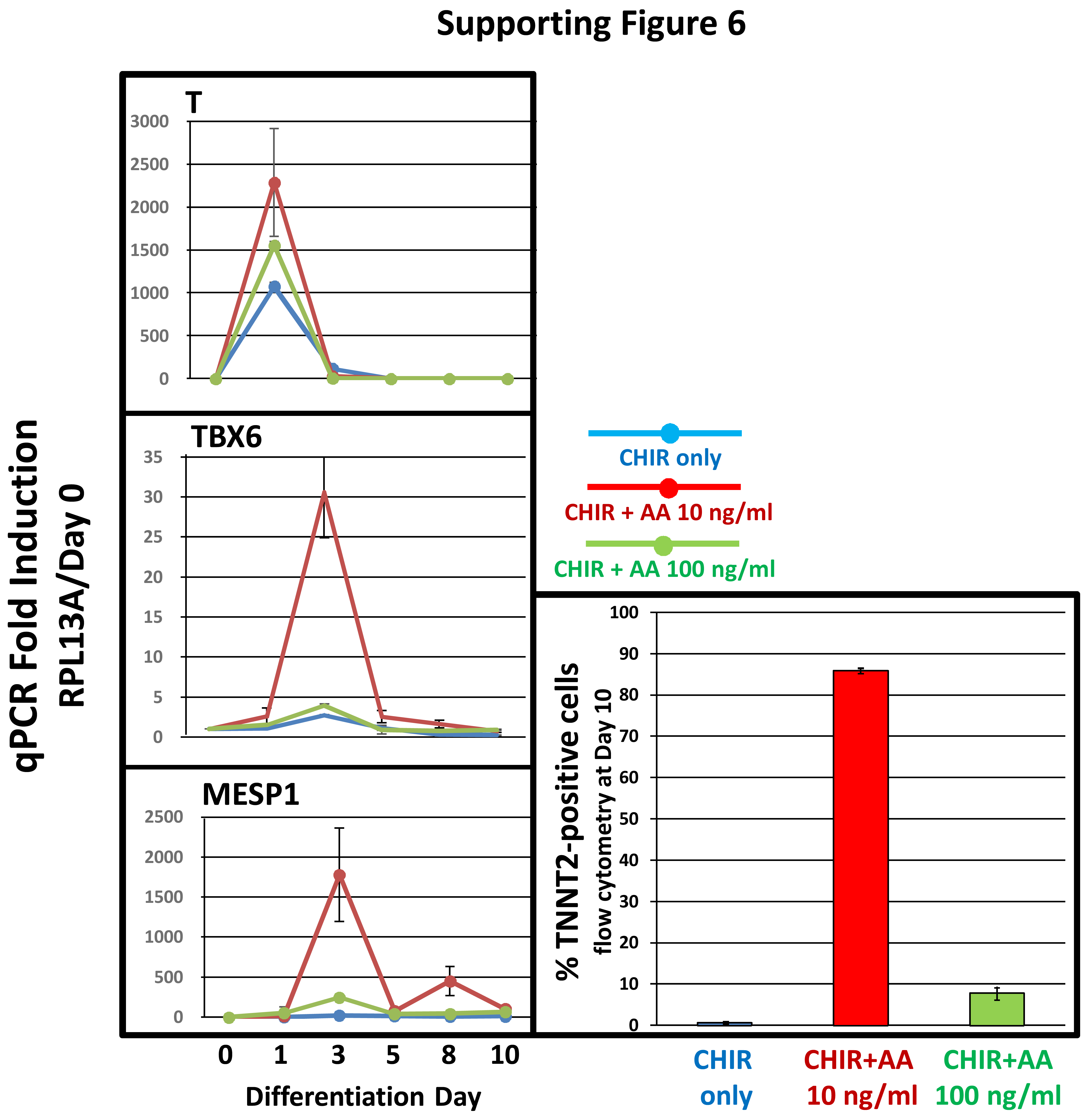

Supplement: S6 Fig — Pluripotent H1 ESCs expanded on Matrigel in mTeSR1 medium were overlaid with Matrigel on Day -1 and induced during Day 0–1 by changing the medium to RPMI/B27 (no insulin) containing CHIR (12 μmol/L) only, CHIR plus 10 ng/ml Activin-A, or CHIR plus 100 ng/ml Activin-A, as indicated. The cultures were treated with IWP (5 μmol/L) during Days 3–5, and, insulin (4,000 ng/ml) was included after Day 7. Left Panels: qPCR-based expression of T (Brachury), TBX6, and MESP1 on the indicated days after induction, normalized to expression of RPL13A and to the level of each gene’s expression in pluripotent cells (Day 0). Right Panel: Flow cytometric determination of cardiomyogenic cell percentages at Day 10. In this experiment, cells induced with CHIR alone during Day 0–1 did not contract at any time, whereas cells induced with CHIR plus 10 ng/ml Activin-A began to rhythmically contract in localized areas at Day 6, which became widespread by Day 10. This determination was unusual in that cultures treated with CHIR plus 100 ng/ml Activin-A during Day 0–1 exhibited localized foci of contracting cells at Day 10. Vertical lines denote ranges of duplicate values; AA = Activin-A. (TIF) [file pone.0118670.s006.tif]

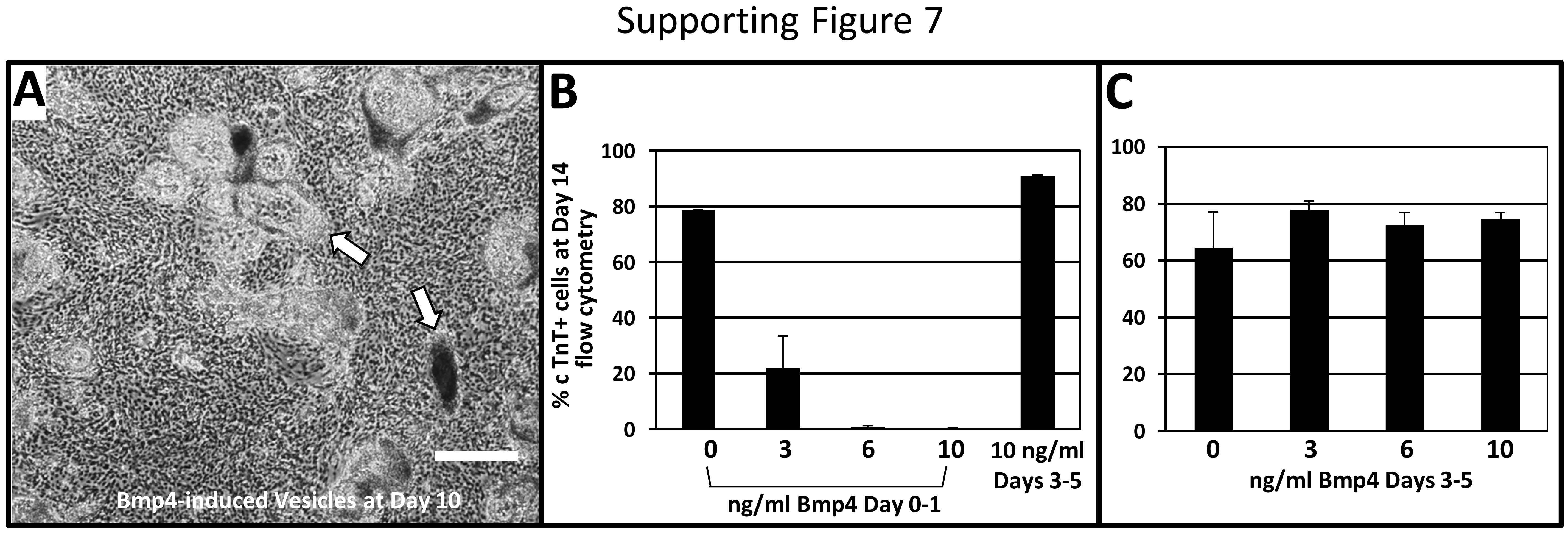

Supplement: S7 Fig — Pluripotent H1 ESCs were induced with CHIR and Bmp4 at the indicated concentrations/durations. Panel A shows Bmp4-induced three-dimensional vesicles (arrows) that begin to appear at Day 7. Panels B-C, percentages of cTnT-positive cells at Day 14. Panel B shows the effect of various Bmp4 concentrations applied during Day 0–1; the effect of treatment with 10 ng/ml during Days 3–5 is shown at right for comparison. Panel C shows the effect of various Bmp4 concentrations during Days 3–5. Vertical lines indicate the range of duplicate values in B, and ±SEM of triplicate values in C. The size bar in A = 200 μm. (TIF) [file pone.0118670.s007.tif]

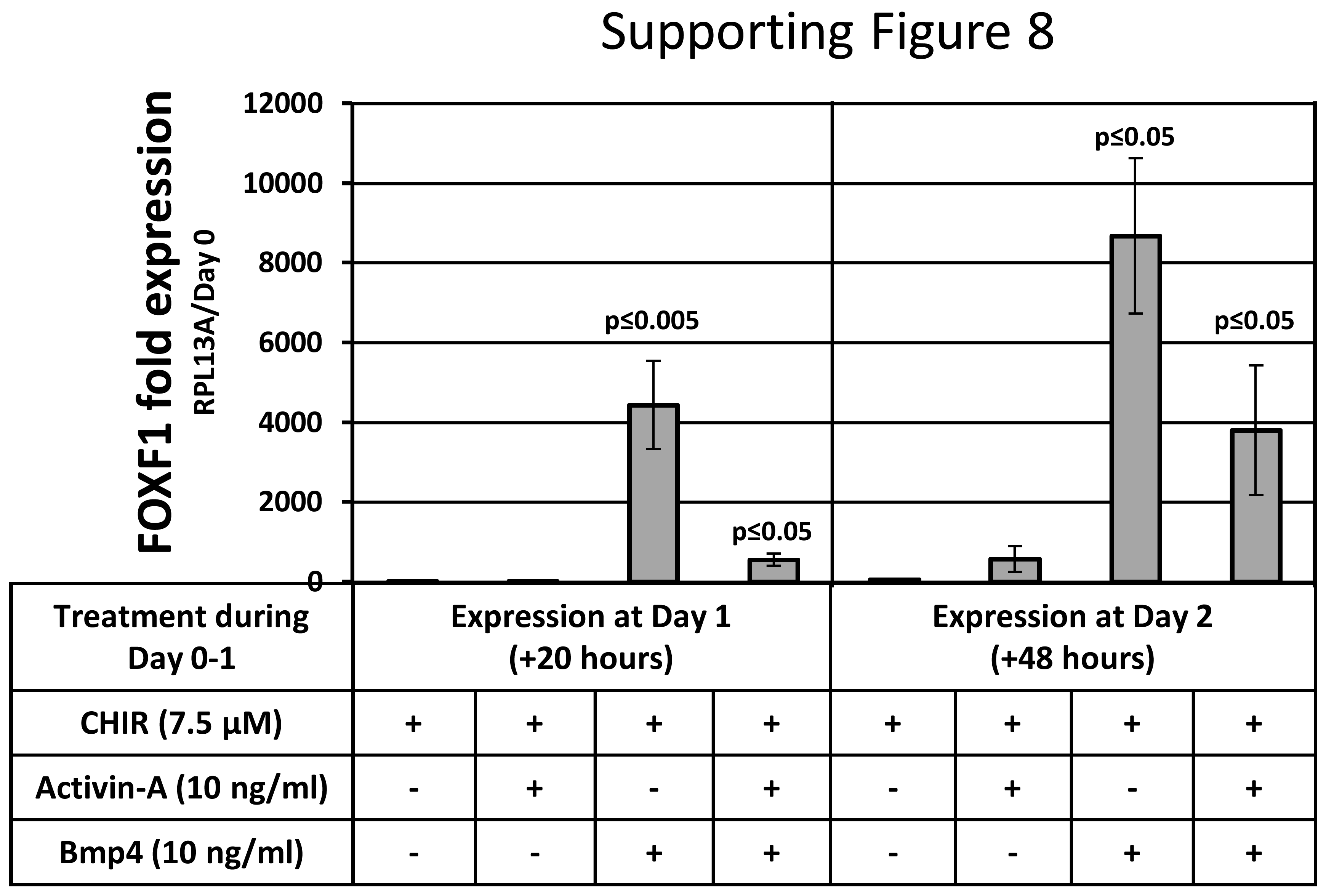

Supplement: S8 Fig — Pluripotent H1 ESCs were induced by changing medium to RPMI/B27 (without insulin) including the indicated factors during Day 0–1. Fold expression of FOXF1 (Y axis) was assessed qRT-PCR and normalized to RPL13A (loading control), and to the levels of these mRNAs in pluripotent cells at Day 0. Numbers in parentheses indicate numbers of cultures; bars/vertical lines indicate mean/±SEM. The p-values are relative to cells treated with CHIR alone. (TIF) [file pone.0118670.s008.tif]

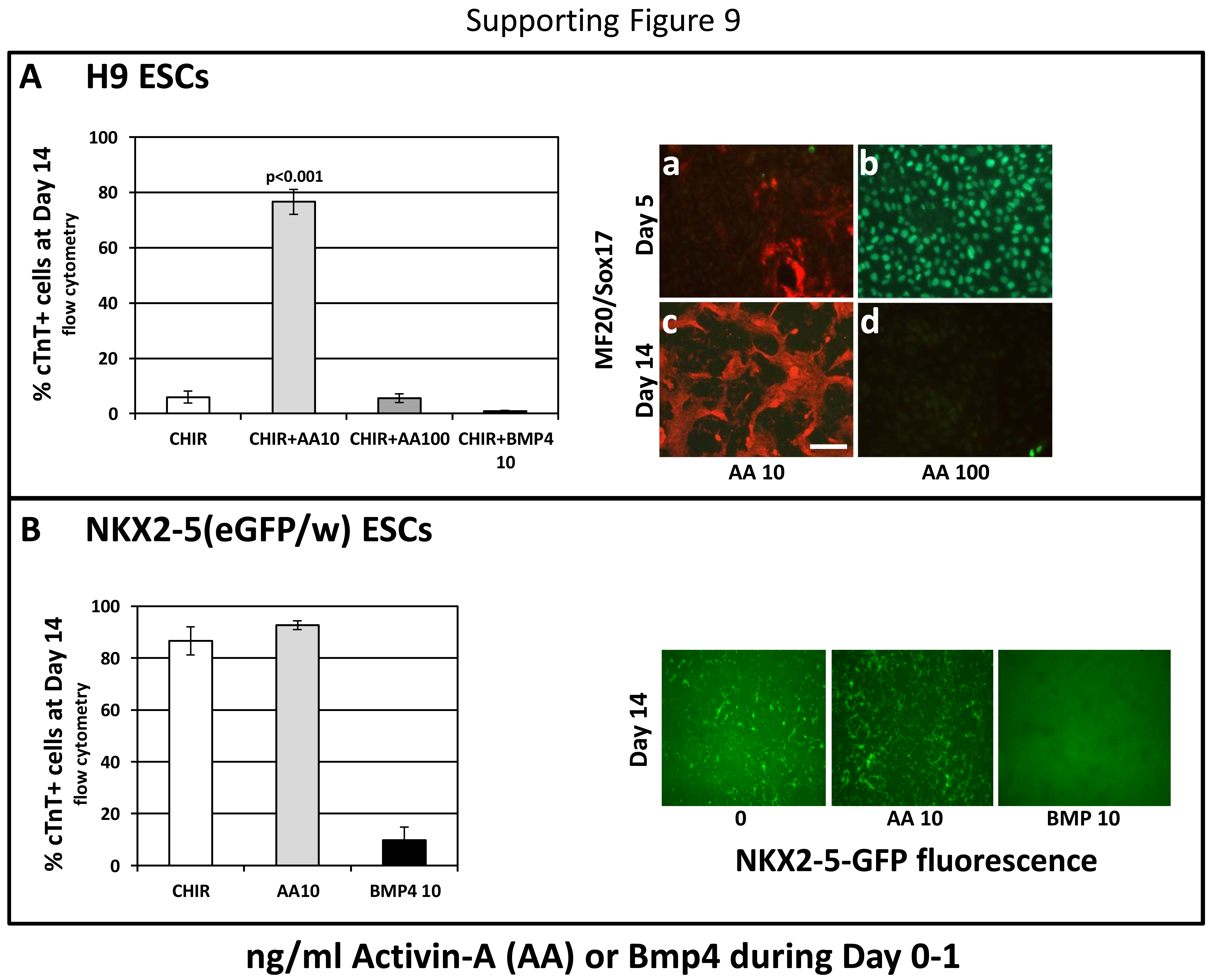

Supplement: S9 Fig — H9 (Panel A) and NKX2-5(eGFP/w) (Panel B) ESCs were expanded and induced with CHIR, along with the indicated levels of Activin-A or Bmp4 during Day 0–1. Panel A: Inclusion of 10 ng/ml Activin-A during induction augmented induction by CHIR alone, the latter of which was relatively ineffective in this determination. Inclusion of 10 ng/ml Bmp4 or 100 ng/ml Activin-A inhibited cardiomyogenesis at Day 14; by Day 5, 100 ng/ml Activin-A induced DE, indicated by Sox17-positive cells (sub-panel b). Red fluorescence = αMHC (MF20) immunostaining; green fluorescence = Sox17 immunostaining. Bars represent the mean of triplicate determinations; vertical lines = ±SEM. The p-value is relative to cells induced with CHIR alone. Panel B: Each condition was evaluated in duplicate cultures. In this experiment, although 10 ng/ml Activin-A did not significantly improve the level of differentiation induced by CHIR alone (which was robust in this instance), co-induction with higher Activin-A levels caused cell death by Day 14 (not shown). Co-induction with 10 ng/ml Bmp4 inhibited CHIR-induced cardiomyogenesis. (TIF) [file pone.0118670.s009.tif]

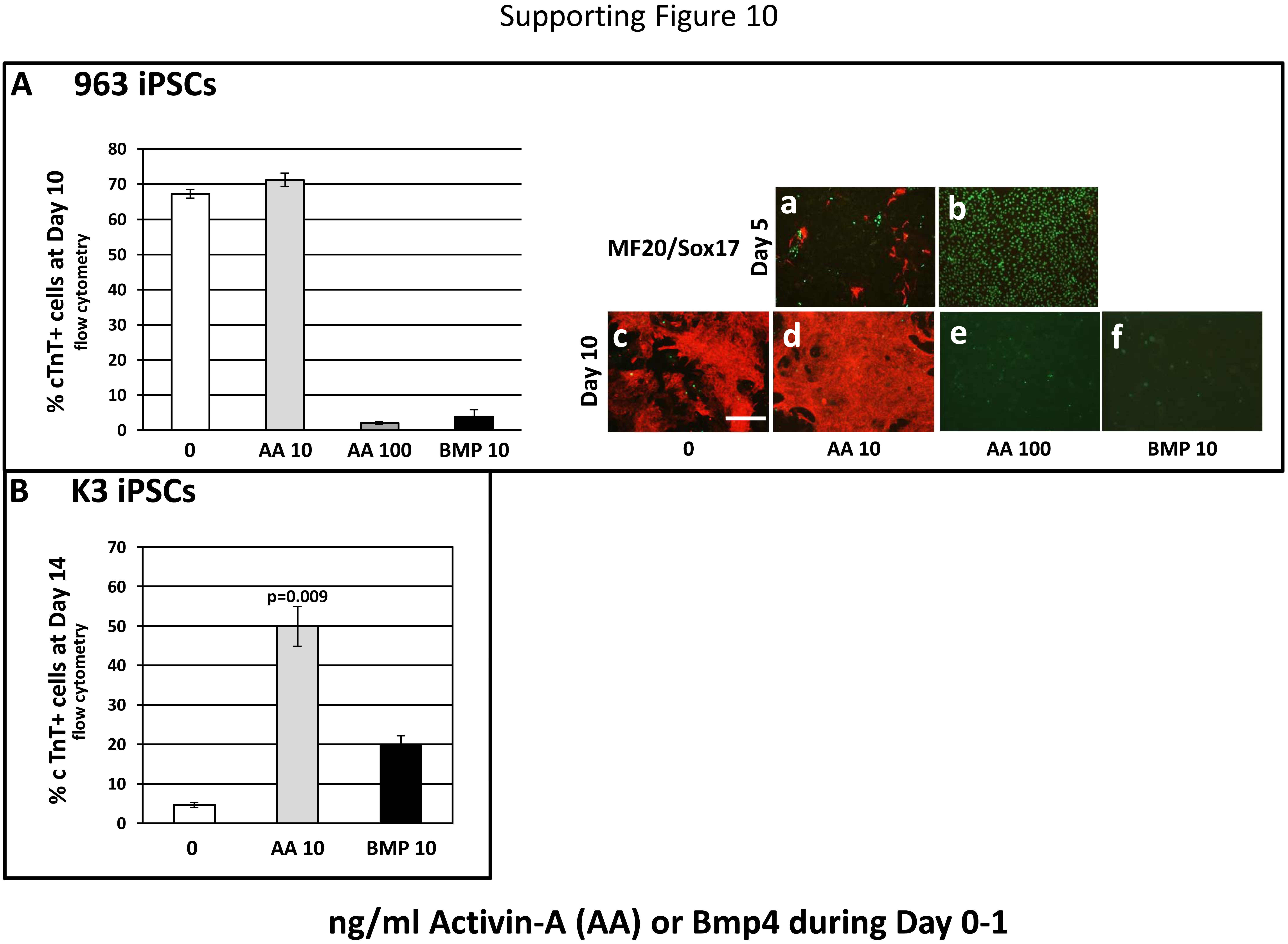

Supplement: S10 Fig — Pluripotent 963 (Panel A) and K3 (Panel B) iPSCs were expanded and induced with CHIR, along with the indicated levels of Activin-A or Bmp4 during Day 0–1. Panel A: Although 10 ng/ml Activin-A did not significantly improve the already high level of differentiation (cTnT-positive cells) induced by CHIR alone in this experiment, co-induction with 100 ng/ml Activin-A inhibited cardiomyogenesis as indicated by reduced numbers of cTnT-positive cells and the absence of both beating and myosin heavy chain staining (sub-panel e) at Day 10; the latter was concomitant with a high incidence of Sox17-positive cells noted at Day 5 (sub-panel b). Co-induction with 10 ng/ml Bmp4 during Day 0–1 inhibited CHIR-induced cardiomyogenesis at Day 10. Red fluorescence = αMHC (MF20) immunostaining; green fluorescence = Sox17 immunostaining. In panel B (K3 iPSCs), inclusion of 10 ng/ml Activin-A during induction strongly augmented the effect of CHIR, which was relatively ineffective in this determination, whereas inclusion of 10 ng/ml Bmp4 inhibited cardiomyogenesis. In both panels, bars represent the mean of triplicate determinations; vertical lines = ±SEM. P values were calculated by Student’s t-test; P values are relative to cells induced with CHIR alone. (TIF) [file pone.0118670.s010.tif]
