# Supplementary material for: Activin-A and Bmp4 Levels Modulate Cell Type Specification during CHIR-Induced Cardiomyogenesis
Source: PLoS One. 2015 Feb 23;10(2):e0118670. doi: 10.1371/journal.pone.0118670 (PMC4338295; doi:10.1371/journal.pone.0118670)
Supplement: S1 Methods — (DOCX) [file pone.0118670.s011.docx]

**SUPPORTING INFORMATION**

**SUPPORTING MATERIALS & METHODS**

**REAGENTS & CELLS**

**Growth Factors & Inhibitors**

Activin-A (338-AC-005), Bmp4 (314-BP-010), Dkk1 (5439-DK-010), Wnt3a (5036-WN-010) were purchased all from R&D Systems. Fgf2 (bFgf) was from Invitrogen (PHG0026). Small molecular weight organic inhibitors were purchased as follows: CHIR99021 (Stemgent 04-0004-2), and IWP2 (Tocris 3533).

**Mouse Embryonic Fibroblast (MEF) Feeders**

When required, mitomycin-C-treated MEF feeders were plated on cell culture dishes pre-coated with 0.1% gelatin (Millipore ES-006-B) at a density of 11,000 cells/cm^2^. MEFs were kept in medium consisting of DMEM (Millipore SLM-021-B), 10% FBS (Invitrogen 16000-044), non-essential amino acids (Invitrogen 11140-050) and pen-strep (Invitrogen 15140-122).

**Maintenance & Passaging of Human Pluripotent Cells**

Human embryonic stem cells (hESCs), lines H1 (WA01) and H9 (WA09), were obtained from the **National Stem Cell Bank** (NSCB; WiCell, Madison WI). Human NKX2-5(eGFP/w) ESCs were a gift from Professors A.G Elefanty and E.G. Stanley, Monash University [1]. Human induced pluripotent cell-line iPSK3 (K3 cells) was re-programmed from foreskin fibroblasts (ATCC cell line CRL2097) via transient transfection with a tandem vector encoding Oct4, Nanog, Sox2 and Lin28 as previously described [2]. Human induced pluripotent cell-line 963 was induced from dermal fibroblasts using the CytoTuneTM-iPS Reprogramming (Invitrogen A1378001) kit, which contains non-integrating, “traceless”, Sendai virus encoding re-programming factors Oct3⁄4, Sox2, Klf4, and cMyc, in accordance with Children’s Hospital of Wisconsin (CHW) and Mayo Medical School IRBs, in partnership with the Wanek Consortium for HLHS (WCH).

Pluripotency was maintained by plating cells in monolayer culture at a density of 125,000/cm^2^ in mTeSR medium (Stem Cell Technologies #05870) on 35 mm dishes that had been pre-coated for 60 minutes at 37 ^o^C with growth factor-reduced Matrigel (8 µg/cm^2^) according to the manufacturer’s recommendations (BD Biosciences #356230). Pluripotent cells were maintained under hypoxic conditions (4% O_2_); medium was changed daily. Upon attaining 75% confluence (every 3-4 days), cells were passaged by incubating in enzyme-free cell dissociation buffer (Millipore #S-014-B) for 8 minutes, followed by gentle trituration and re-plating at a 1:6 split on Matrigel-coated dishes.

**Antibodies & Immunostaining**

Antibodies used for immunofluorescent staining included anti-Myosin Heavy Chain monoclonal (Developmental Studies Hybridoma Bank MF20); anti-sarcomeric α-Actinin mouse monoclonal (Abcam Ab9465), anti-OCT4 monoclonal (Chemicon MAB-4305) or rabbit polyclonal (Santa Cruz sc-9081); and anti-SOX17 goat polyclonal (R&D Systems AF1924). To perform immunohistochemistry cells were rinsed with PBS, followed by 15 min fixation with fresh 4% paraformaldehyde, 15 min incubation with 0.5% Triton-X-100, and 30 min blocking with 3% BSA/PBS. Primary antibodies were applied in 1% BSA/PBS overnight. After washing, the secondary antibodies – donkey anti-rabbit Alexa Fluor 488 (Invitrogen A21206) or donkey anti-goat Alexa Fluor 568 (Invitrogen A11057) – were applied in 1% BSA/PBS (1:1,000) for 1 hr. Nuclei were stained with DAPI.

Antibodies for flow cytometry included mouse monoclonal anti-cardiac Cardiac Troponin T (cTnT, TNNT2; Thermo Scientific MS-295-R7) followed by secondary antibody goat anti-mouse 594 IgG1 (y1; Invitrogen #A21125). Alexa Fluor 488-conjugated mouse myeloma IgG MOPC-21 was used as an IgG1 isotype control (Thermo Scientific 557721).

**DIFFERENTIATION USING GROWTH FACTORS**

**Pluripotent Phase**

The experiments described in Figures S1 and S2 used pluripotent cells maintained on E-cadherin substrate (StemAdhere, Stem Cell Technologies 07170; 10 µg/ml in PBS with Ca^++^ & Mg^++^). E-cadherin was applied to p60 Petri plates in a volume of 1.5 ml (~0.7 µg/cm^2^) for one hour at 37 ^o^C, followed by aspiration. Pluripotent cells were plated in hESC medium containing freshly added Fgf2 (4 ng/ml) that had been pre-conditioned by MEFs for the preceding 24 hour period (i.e. MEF-conditioned medium = MEF-cm). Pluripotent cells were passaged when nearly confluent (5-7 days) by aspirating medium and rinsing with PBS without calcium and magnesium, followed by brief incubation in the same buffer at room temperature. Just prior to cellular detachment (approx. 8 minutes), PBS was replaced with MEF-cm, followed by immediate trituration and re-plating at a 1🡪4 split on dishes coated with E-cadherin (13,000-14,000 cells/cm^2^). After monitoring >70 passages, no loss of Oct4 immunostaining or expression of differentiation gene markers was noted.

**Differentiation Phase**

For the experiments described in Figures S1 and S2, differentiation was induced in pluripotent cells that had been sub-cultured from E-cadherin onto Matrigel substrate. Matrigel-coated plates were prepared by diluting Matrigel (BD Biosciences, hESC-qualified, 354277) to 50 µg/ml using ice-cold DMEM/F12, then adding 1.0 ml to each well of a 12-well plates (Corning 3513) for one hour at room temperature, followed by aspiration. Pluripotent cells were dissociated from E-cadherin with Accutase (Invitrogen A11105-01), counted in a Guava EasyCyte™ Mini Base System (Millipore 0500-1430) and adjusted to a concentration of 1.0 x 10^6^/ml by adding MEF-cm, followed by plating 1.0 ml in each well of a Matrigel-coated 12-well plate (~250,000 cells/cm^2^). Cells were kept pluripotent on Matrigel for 3 days under hypoxic (4% O_2_) conditions to attain confluence, followed by growth for two additional days to achieve high cellular density. MEF-cm was replaced daily. On Day 0, differentiation was induced by exchanging MEF-cm for RPMI Medium 1640 (Invitrogen 11835-030) supplemented with B27 (Invitrogen 0050129SA) without insulin, plus pen-strep, non-essential amino acids, and growth factors as indicated. Medium was exchanged daily.

**DIFFERENTIATION USING THE 2-INHIBITOR PROTOCOL**

**Pluripotent Phase**

Pluripotent cells were induced into cardiomyocytes as recently described [3]. Briefly, pluripotent cells were dissociated without enzymes and re-plated at high density (~ 95% confluence) in an hypoxic environment (4% O_2_) on 35 mm culture dishes; this was designated as Day -4 of the protocol shown in Figure 1. Note that maintenance of pluripotent cells under hypoxia was the only change from the original published protocol [4]. After approximately four days, during which time mTeSR1 medium (1.5 ml) was replaced daily, the cultures attained super-confluency (~125%). As indicated (Fig. 1), Matrigel (8 µg/cm^2^) was re-applied with the medium change at Day -3 or Day -1.

**Differentiation Phase**

On Day 0, differentiation of super-confluent cells was induced by moving the cultures to a normoxic environment and changing the medium to 1.5 ml RPMI/B27 without insulin, with CHIR99021 (Stemgent 04-0004-2) at a concentration of 12 µmol/L (lot #2721) or of 7.5 µmol/L (lot #2914); in our experience, efficacy of CHIR is lot-dependent. After 21 hours (Day +1), when approximately 25% of the cells become detached, the medium was replaced with RPMI/B27 medium without insulin and without CHIR, using a volume of 2.0 which we have noted to improve outcomes. Medium was not changed again until Day +3, when it was replenished with 2.0 ml RPMI/B27 without insulin, with 5 µmol/L IWP (Tocris 3533). Two days later (Day +5) the medium was replaced with RPMI/B27 without insulin and without inhibitors. At Day +7, the medium was changed to 2.0 ml RPMI/B27 with insulin, followed by identical medium changes at 2 day intervals thereafter.

**Monitoring Cardiomyocyte Differentiation**

Cultures were carefully monitored for the onset of rhythmic beating. At Day +14, experiments were terminated and cells were processed for evaluation of percentages of cardiac troponin-T-positive (cTnT+) cells via flow cytometry, which was performed in duplicate dishes and correlated with immunostaining of α-myosin heavy chain (αMHC; MF-20) in a parallel culture.

**QUANTITATIVE RT/PCR**

For the qPCR determination described in Figure S2, approximately one-half of the cells in each culture well were harvested by scraping on the indicated days and RNA was immediately purified using RNeasy Plus Mini Kit (Qiagen 74134), quantitated by absorption at A_260_, and stored at -80 ^o^C. Reverse-transcription (RT) was performed using the Bio-Rad iScript cDNA Synthesis Kit (170-8891); within each experiment, identical quantities of RNA from each sample were reverse-transcribed (250-1,000 ng, depending on the experiment). For realtime PCR, 10% of each RT product was used as template by suspending in RT2 SYBR Green/Fluorescein qPCR Master Mix (SABiosciences #PA-011-24) in a final volume of 25 µl, followed by amplification using a Bio-Rad iCycler with a hot start followed by alternating steps of annealing/elongation (60 sec @ 60 ^o^C) and denaturation (15 sec @ 95 ^o^C). Reactions were performed in 96-well SABiosciences Custom Profiler Array plates in which the following primer pairs were embedded in individual wells: POU5F1 (PPH02394); NANOG (PPH17032), T (brachury, PPH02753), PAX6 (02598), SOX17 (PPH 02451), MESP1 (PPH21511), ISL1 (PPH02461), NKX2.5 (PPH 02462), TBX5 (PPH06918), TNNT2 (PPH 02619), MYH6 (PPH02439), and GUSB (PPH01096) or RPL13A (PPH0102B) as loading controls. As required, individual qPCR reactions were carried out in 25 μl final volume that included 12.5 μl RT2 qPCR master mix (SABiosciences PA-011), 10.5 μl nuclease-free DW, 1.0 μl cDNA template, and 1.0 μl of a qPCR-certified primer pair.

The qPCR determination described in Figure S6 was similarly performed, except that the primer pairs were purchased from Bio-Rad (#100-25636) and that qPCR was performed using the PrimePCR SYBR Green Assay according the manufacturer’s instructions.

**RNA-SEQ**

Total RNA, free from genomic DNA contamination, was extracted from confluent 35 mm dishes of H1 ESCs on Days 0, 1, 3, 5, 8 and 14 using the Qiagen RNeasy Plus Mini Kit (Valencia, CA). All samples had RNA Integrity Numbers ≥9.40 as determined by Bioanalyzer 2100 (Agilent Technologies, Santa Clara, CA). Using the TruSeq kit version 2.5 (Illumina, San Diego, CA), RNA sequencing libraries were prepared using 500 ng total RNA, spiked with Ambion® ERCC external RNA controls according to the manufacturer’s specifications (Life Technologies, Carlsbad, CA). Briefly, magnetic bead technology was used to isolate and elute mRNA, which was primed and fragmented to sizes of approximately 300 bp. First- and second-strand cDNA synthesis was performed using Superscript II reverse transcriptase (Invitrogen, Carlsbad, CA). After end-repair and A-tailing reactions, unique indices were introduced according to the protocol in order to enable sample multiplexing during the sequencing run. qPCR-quantitation of the library and sequencing services were performed on an Illumina HiSeq 2000 platform by the Sequencing Core of the Human and Molecular Genetics Center (Medical College of Wisconsin). Running two samples per lane generated ~90 million paired reads per sample.

Reads were mapped to the human genome (NCBI build 37) using gapped alignment software Bowtie 2.0.0.6 and Tophat 2.0.0, which together accurately aligned an average of 99% of paired-end reads [5]. Non-standard parameters for Tophat were used to increase mappability, including increasing sequence mismatch count to 3, and maximum indel size to 5. Maximum intron length was 75 kilobases. Resulting BAM files were further filtered with Picard 1.62 ([http://picard.sourceforge.net](http://picard.sourceforge.net/)) to remove reads having apparent fragment insert sizes over twenty kilobases, because reads this size have been primarily found on mis-mapped pairs bridging homologous regions. Reads were streamed from the filtered BAM using Samtools0.1.18 to count only those reads where both pair ends were mapped concordantly [6]. Differential expression was computed for whole gene regions by summing reads for each region (as defined by the UCSC Ref-Seq database) with coverageBED 2.14.3 from the bedtools package [7]. Because the Ref-Seq database contains entries for each exon of a gene, these were summed with a SQLite 3.6.20 application. The final counts table consists of integer-valued aligned paired read measurements at 37,857 unique NM_* and NR_* identifiers per sample. This table was processed using DESeq 1.6.1 to generate differential expression lists [8]. Assigning one transcript as representative of each named gene yielded 18,851 expression measurements per sample.

**SUPPORTING REFERENCES**

1. Elliott DA, Braam SR, Koutsis K, Ng ES, Jenny R, et al. (2011) NKX2-5(eGFP/w) hESCs for isolation of human cardiac progenitors and cardiomyocytes. Nat Methods 8: 1037-1040.

2. Si-Tayeb K, Noto FK, Sepac A, Sedlic F, Bosnjak ZJ, et al. (2010) Generation of human induced pluripotent stem cells by simple transient transfection of plasmid DNA encoding reprogramming factors. BMC Dev Biol 10: 81.

3. Lian X, Zhang J, Azarin SM, Zhu K, Hazeltine LB, et al. (2012) Directed cardiomyocyte differentiation from human pluripotent stem cells by modulating Wnt/beta-catenin signaling under fully defined conditions. Nat Protoc 8: 162-175.

4. Lian X, Hsiao C, Wilson G, Zhu K, Hazeltine LB, et al. (2012) Robust cardiomyocyte differentiation from human pluripotent stem cells via temporal modulation of canonical Wnt signaling. Proc Natl Acad Sci U S A 109: E1848-1857.

5. Trapnell C, Pachter L, Salzberg SL (2009) TopHat: discovering splice junctions with RNA-Seq. Bioinformatics 25: 1105-1111.

6. Li H, Handsaker B, Wysoker A, Fennell T, Ruan J, et al. (2009) The Sequence Alignment/Map format and SAMtools. Bioinformatics 25: 2078-2079.

7. Quinlan AR, Hall IM (2010) BEDTools: a flexible suite of utilities for comparing genomic features. Bioinformatics 26: 841-842.

8. Anders S, Huber W (2010) Differential expression analysis for sequence count data. Genome Biol 11: R106.
